# Investigational Drugs for the Treatment of Depression (Part 1): Monoaminergic, Orexinergic, GABA-Ergic, and Anti-Inflammatory Agents

**DOI:** 10.3389/fphar.2022.884143

**Published:** 2022-06-14

**Authors:** Octavian Vasiliu

**Affiliations:** Department of Psychiatry, Dr.Carol Davila University Emergency Central Military Hospital, Bucharest, Romania

**Keywords:** treatment-resistant depression, brexanolone, immunomodulators, orexin receptors antagonists, triple monoamines reuptake inhibitors, atypical antipsychotics

## Abstract

Therapeutic management of depression has currently important limitations, and its low efficacy is reflected in high rates of non-response even after multiple trials of antidepressants. Almost two-thirds of the patients diagnosed with major depression who received a 4–6 weeks trial of antidepressant could not reach remission, and more than 30% of these patients are considered treatment-resistant. In bipolar depression, the situation is also discouraging if we analyze the high suicide rate, the risk for the treatment-emergent affective switch when antidepressants are added, the high rate of treatment resistance (up to 25%), and the severe functional impairments associated with these episodes. Therefore, new therapeutic agents are needed, as well as new pathogenetic models for depression. The vast majority of the currently approved antidepressants are based on the monoamine hypothesis, although new drugs exploiting different neurotransmitter pathways have been recently approved by FDA. Brexanolone, an allopregnanolone analog, is an example of such new antidepressants, and its approval for post-partum depression inspired the search for a new generation of neurosteroids and GABA-ergic modulators, with an easier way of administration and superior tolerability profile. Orexin receptors antagonists are also extensively studied for different psychiatric disorders, depression included, in phase II trials. Antiinflammatory drugs, both cyclo-oxygenase 2 inhibitors and biological therapy, are investigated in patients with depressive disorders based on the proven correlation between inflammation and mood disorders in preclinical and clinical studies. Also, a new generation of monoamine-based investigational drugs is explored, ranging from triple reuptake inhibitors to atypical antipsychotics, in patients with major depression. In conclusion, there is hope for new treatments in uni- and bipolar depression, as it became clear, after almost seven decades, that new pathogenetic pathways should be targeted to increase these patients’ response rate.

## Introduction

The exploration of therapeutic options for major depressive disorder (MDD) is very important for clinicians, due to the significant functional impairment, high rate of relapse, and treatment resistance associated with this pathology (de Sousa et al., 2015; Lacerda, 2020). The worldwide prevalence of MDD is estimated to be around 16%, and remission is obtained by only one-third of these patients (de Sousa et al., 2015; Lacerda, 2020). The pathophysiology of MDD is still largely unknown, and the monoamine hypothesis remains the most explored explanation supported by data from animal models and human trials (de Sousa et al., 2015). Although large efforts have been invested in the research of neurobiologically oriented treatments for MDD, only a few products have been FDA-approved outside the conceptual framework of the monoamine hypothesis. These exceptions are brexanolone, for post-partum depression, and esketamine for treatment-resistant MDD. However, multiple pathogenetic mechanisms have been investigated, from dysfunctions in the orexinergic or γ-aminobutyric acid (GABA) neurotransmission to glutamatergic, opioidergic, or sestrin modulators (Schüle et al., 2014; Poleszak et al., 2016; Fogaça et al., 2019; Sengupta et al., 2019; Han et al., 2020). A new generation of monoaminergic agents has been studied, e.g. new triple monoaminergic inhibitors and new atypical antipsychotics (Fava et al., 2019; Mi et al., 2021). Old drugs have been repurposed as antidepressants or adjuvants to current antidepressant treatment, in the hope of finding new ways to mitigate residual symptoms or to increase the chance of reaching a response/remission in treatment-resistant MDD patients (Vasiliu et al., 2017; Kalmoe et al., 2020; Papakostas et al., 2020). Combining different classes of pharmacological agents in one formulation is another explored strategy to increase the potency of the antidepressant treatment. This approach is based on reciprocal augmentation of different drugs’ pharmacodynamical properties, mitigating the risk of certain adverse events to one drug by adding another, or exploiting their distinct pharmacokinetic properties to increase the plasma concentration of a specific agent (Thase et al., 2019; Sakurai et al., 2022).

The limited efficacy of currently marketed antidepressants is only one of the challenges that clinicians are facing, another important aspect of the therapeutic management being the low tolerability profile of several drugs, pharmacokinetic interactions at CYP450 isoenzymes with concomitantly administered medications for comorbid disorders, long duration until the antidepressant effect onset, the necessity of long-term drugs administration, *etc*. (Vasile et al., 2011; Vasiliu, 2019).

Therefore, this review has as its main objective to explore new investigational products with antidepressant properties, considering their phase of development, their reported efficacy and tolerability, and their contribution to the construction of a new pathogenetic model of depression.

## Methodology

A systematic review of the papers referring to new drugs in different phases of clinical research was conducted through the main electronic databases (PubMed, MEDLINE, Cochrane, Web of Science (Core Collection), PsychINFO, Scopus, and EMBASE using the paradigm “investigational antidepressants/products” OR “new antidepressants/agents” AND “clinical trial” AND “major depressive disorder” OR “bipolar disorder” OR “depression.” Lists of references for every article corresponding to the search paradigm were investigated, and they were added to the review if they were not detected through the previously mentioned paradigm.

A broad search was chosen to include the widest variety of molecules corresponding to the review’s objective. For this purpose, a supplementary search was added, targeting investigational products for depression explored in the clinical trials repositories run by the United States National Library of Medicine and the National Institutes of Health (clinicaltrials.gov), World Health Organisation (International Clinical Trials Registry Platform), and European Union (EU Clinical Trial Register). The search within the clinical trial databases was structured by the disorder- “depression” (both unipolar and bipolar), type- “interventional,” population- “adults” and “adolescents,” and trial phase from I to III, but all statuses of recruitment were allowed. If the outcome of a registered trial for an investigational product was not mentioned in any of the explored repositories, the respective drug manufacturer’s site was explored, to verify if any results are available.

All papers and references from the previously mentioned electronic databases were allowed in the primary search, if they were published between January 2000 and February 2022. Data regarding clinical studies found in the repositories were also included in the primary search.

**FIGURE 1:**
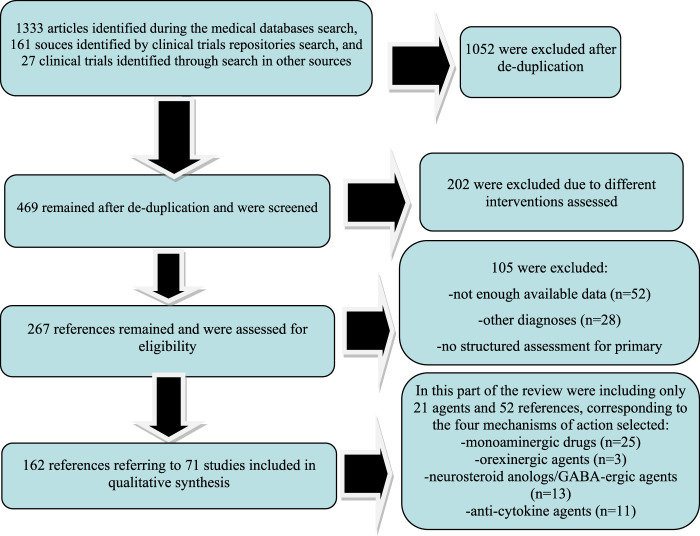
Results of the PRISMA-based search paradigm.

This systematic review is based on the Preferred Reporting Items for Systematic Reviews and Meta-Analyses (PRISMA) statement, and all the data collection, review, reporting, and discussion were conducted according to this statement (Figure 1) (Moher et al., 2009). The inclusion and exclusion criteria are presented in Table 1. The PRIMA-P Checklist (Moher et al., 2015) is presented in Table 2.

**TABLE 1:** Inclusion and exclusion criteria.

Operational criteria	Inclusion criteria	Exclusion criteria
Population	Selected population groups were allowed—adolescents and adults. No superior age limit was specified. The main diagnoses were major depressive disorder and bipolar depression. Treatment-resistant forms, first mood episodes, or chronic depression were included. Chronic organic co-morbidities were allowed. Diagnoses should be based on criteria specified by the authors of that paper/sponsors of the trial. Both ICD10 and DSM (IV, IV-TR, or 5) diagnosis criteria were allowed	Studies that did not specify age limits for their samples, and studies that enrolled children. The presence of psychiatric comorbidities with significant impact on cognition, mood, behavior, and overall functionality (e.g., psychotic disorders, severe neurocognitive disorders, substance use disorders)
Intervention	Pharmacological, or combined, pharmacological and psychotherapeutic interventions. New investigational drugs, or repurposed drugs for antidepressant use, were included. Only monoaminergic, orexinergic, GABA-ergic/neurosteroids, and anti-inflammatory agents are included in this part of the review	Psychotherapy as monotherapy for MDD/bipolar depression. Already marketed antidepressants, FDA-approved for all the indications specified in the “population” section of this table, if they were the main intervention. These types of agents were allowed only as active comparators
Environment	Both in-patient and out-patient regimens	Unspecified environment
Primary and secondary variables	Evaluation of the efficacy, safety, and tolerability of new investigational drugs with antidepressant properties	All research with unspecified variables. Reviews without predefined quantifiable objectives, or poorly defined primary outcome measures
Study design	Any phase of clinical investigation from I to III was admitted if it corresponded to the predefined objective of this review. Phase IV studies were permitted, if specific variables related to depression were included, for drugs not approved for this indication	Studies with unspecified or poorly defined design. Studies with unclearly defined population/statistical methods. Case reports, case series
Language	Any language of publication was admitted if the *in-extenso* published paper was available. The same language criteria were applied for clinical trials identified in metadata repositories	—

**TABLE 2:** PRISMA-P 2015 Checklist (Moher et al., 2015). This checklist has been adapted for use with protocol submissions to Systematic Reviews from Table 3 in Moher D et al: Preferred reporting items for systematic review and meta-analysis protocols (PRISMA-P) 2015 statement. Systematic Reviews 2015 4:1.

Section/topic	#	Checklist item	Information reported		Line number(s)
			Yes	No	
**Administrative information**					
Title					
Identification	1a	Identify the report as a protocol of a systematic review	⊠	□	68–74
Update	1b	If the protocol is for an update of a previous systematic review, identify it as such	□	□	Not applicable
Registration	2	If registered, provide the name of the registry (e.g., PROSPERO) and registration number in the Abstract	□	□	Not applicable
Authors					
Contact	3a	Provide the name, institutional affiliation, and e-mail address of all protocol authors; provide the physical mailing address of the corresponding author	⊠	□	4–9
Contributions	3b	Describe contributions of protocol authors and identify the guarantor of the review	□	□	Not applicable, only one author
Amendments	4	If the protocol represents an amendment of a previously completed or published protocol, identify it as such and list changes; otherwise, state a plan for documenting important protocol amendments	□	□	Not applicable
Support					
Sources	5a	Indicate sources of financial or other support for the review	⊠	□	755
Sponsor	5b	Provide a name for the review funder and/or sponsor	□	□	Not applicable
Role of sponsor/funder	5c	Describe roles of funder(s), sponsor(s), and/or institution(s), if any, in developing the protocol	□	□	Not applicable
**Introduction**					
Rationale	6	Describe the rationale for the review in the context of what is already known	⊠	□	14–63
Objectives	7	Provide an explicit statement of the question(s) the review will address concerning participants, interventions, comparators, and outcomes (PICO)	⊠	□	64–66, Table 1
**METHODS**					
Eligibility criteria	8	Specify the study characteristics (e.g., PICO, study design, setting, time frame) and report characteristics (e.g., years considered, language, publication status) to be used as criteria for eligibility for the review	⊠	□	68–96, Table 1
Information sources	9	Describe all intended information sources (e.g., electronic databases, contact with study authors, trial registers, or other grey literature sources) with planned dates of coverage	⊠	□	69–70, 77–80
Search strategy	10	The present draft of the search strategy is to be used for at least one electronic database, including planned limits, such that it could be repeated	⊠	□	68–96
*Study records*					
Data management	11a	Describe the mechanism(s) that will be used to manage records and data throughout the review	⊠	□	92–96
Selection process	11b	State the process that will be used for selecting studies (e.g., two independent reviewers) through each phase of the review (i.e., screening, eligibility, and inclusion in meta-analysis)	⊠	□	68–74, Table 1
Data collection process	11c	Describe the planned method of extracting data from reports (e.g., piloting forms, done independently, in duplicate), and processes for obtaining and confirming data from investigators	⊠	□	82–84
Data items	12	List and define all variables for which data will be sought (e.g., PICO items, funding sources), any pre-planned data assumptions, and simplifications	⊠	□	Table 1
Outcomes and prioritization	13	List and define all outcomes for which data will be sought, including prioritization of main and additional outcomes, with rationale	⊠	□	Table 1
Risk of bias in individual studies	14	Describe anticipated methods for assessing the risk of bias of individual studies, including whether this will be done at the outcome or study level, or both; state how this information will be used in data synthesis	⊠	□	82–84
***DATA***					
Synthesis	15a	Describe criteria under which study data will be quantitatively synthesized	□	⊠	—
	15b	If data are appropriate for quantitative synthesis, describe planned summary measures, methods of handling data, and methods of combining data from studies, including any planned exploration of consistency (e.g., *I* ^2^, Kendall’s tau)			—
	15c	Describe any proposed additional analyses (e.g., sensitivity or subgroup analyses, meta-regression)	□	⊠	—
	15d	If quantitative synthesis is not appropriate, describe the type of summary planned	⊠	□	92–96
Meta-bias(es)	16	Specify any planned assessment of meta-bias(es) (e.g., publication bias across studies, selective reporting within studies)	□	⊠	—
Confidence in cumulative evidence	17	Describe how the strength of the body of evidence will be assessed (e.g., GRADE)	□	⊠	—

All pharmacological agents included in the collected data were grouped into nine categories: monoamine-based drugs, orexin receptors modulators, GABA-A receptors modulators and neurosteroid analogs, anti-inflammatory therapies, glutamatergic antidepressants, sestrin modulators, cholinergic agents, combinations of agents, and a residual category for all other molecules with distinct mechanisms of action. In this part of the review, only the first four categories will be analyzed.

## Results

**FIGURE 2:**
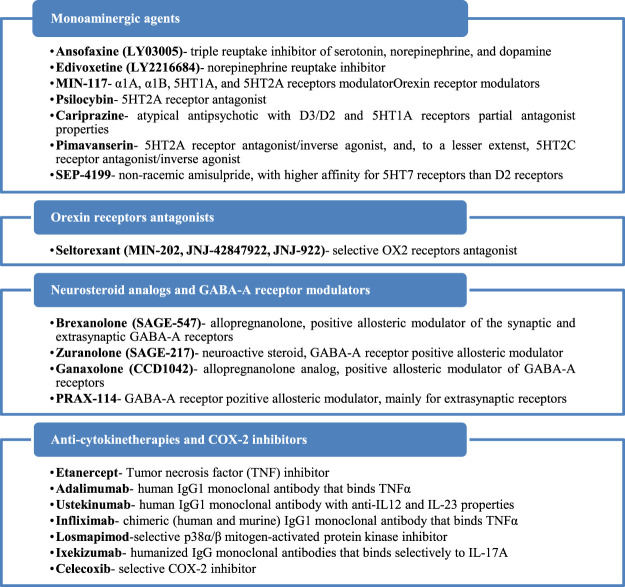
Mechanisms of action of the identified antidepressants in the pipeline, which are presented in this review.

Seven investigational products with a monoamine-based mechanism of action were found in 25 references, including one phase I study, 13 phase II, one phase II/III, and eight phase III trials. One orexin receptor-modulator had been identified in three references, including two phase I studies and one phase II trial. Four neurosteroid analogs or GABA-A receptor modulators have been identified in 13 references, including one phase I study, six phase II, one phase II/III, four phase III, and one not assessed for a clinical phase trial. Eight anti-cytokine therapy and one COX-2 inhibitor have been identified in 11 sources, and their anti-depressive properties have been explored in one phase I study, five phase II, seven phase III, and two phase IV trials. All these agents and their mechanisms of action are presented in Figure 2.

## Investigational Drugs With Monoaminergic Mechanism of Action

In this section different molecules have been included, based on their common property of modulating one or more monoamine systems, classically involved in the pathogenesis of mood disorders (serotonin, dopamine, noradrenaline). *New antidepressants* that possess the ability to inhibit monoamine reuptake, *psilocybin* (which antagonizes 5HT2A receptors), *new atypical antipsychotics* (cariprazine, lumateperone), and *non-racemic amisulpride* (SEP-4199) are analyzed from their efficacy and tolerability perspective in patients with depressive disorders (Table 3).

**TABLE 3:** Monoaminergic modulators with antidepressant properties in the pipeline.

Authors/Trial sponsor	Methodology	Results	Clinical trial phase, trial identifier (if available)	
Mi et al. (2021)	Ansofaxine (LY03005), DBRCT, *N* = 255, MDD, 6 weeks	HAMD-17 total score changes at week 6 were significant vs placebo. The overall tolerability was good		Phase II, NCT03785652
Luye Pharma (2021), NLM (2021a)	Ansofaxine, DBRCT, *N* = 58, MDD, 8 weeks	MADRS total score, HAMD-17 total score, CGI, HAMA, HAMD-17 Anxiety/Somatization factor, Cognitive Impairment factor, Blocking factor, MADRS Anhedonia factor, SDS total score—all were statistically significant improved vs placebo at week 8. No SAE occurred during this trial. Nausea, vomiting, headache, and drowsiness were the most commonly reported adverse events		Phase III, NCT04853407
Ball et al. (2016)	Edivoxetine (LY2216684) adjunctive to the ongoing antidepressant regimen, three DBRCT, *N* = 701, 689, and 449, MDD with partial response to SSRI, 8 weeks	The mean outcome was the mean change from baseline to week 8 in the MADRS total score. This outcome was not reached by any of these three trials. Most of the secondary objectives were not reached, either		Phase III, NCT01173601 Phase III, NCT01187407 Phase III, NCT01185340
Oakes et al. (2015)	Edivoxetine, *N* = 1249, MDD, 8 weeks open-label (edivoxetine + SSRI) + open-label 12 weeks stabilization period + DBRCT 24 weeks	No significant difference between edivoxetine and placebo was detected at the end of the trial (evaluated by MADRS total score)		Phase III, NCT01299272
Ball et al. (2014)	Edivoxetine /placebo adjunctive to SSRI, DBRCT, *N* = 131, MDD partial responsive to SSRI, 10 weeks	No significant differences in efficacy between groups at the end of the trial, based on the MADRS total score		Phase II, NCT00840034
Pangallo et al. (2011)	Edivoxetine, DBRCT, *N* = 495, MDD, 10 weeks	MADRS scores were improved significantly by edivoxetine vs placebo at week 10. Higher rates of response and remission were higher with edivoxetine. SDS scores also were significantly improved vs placebo		Phase II/III, NCT00795821
Ball et al. (2015)	Edivoxetine as adjunctive to SSRI, open-label, *N* = 328, MDD with partial response to SSRI, 54 weeks	The study discontinuation rate due to adverse events was 17%, 13 SAE (1 death). Most commonly reported adverse events: nausea, hyperhidrosis, constipation, headache, dry mouth, dizziness, vomiting, insomnia, upper respiratory tract infection. Mean MADRS score improvements were −17.0 at week 54		Phase III, NCT01155661
Davidson et al. (2016)	MIN-117 vs placebo vs paroxetine, DBRCT, *N* = 84, moderate-to-severe MDD, 6 weeks	MADRS total score was improved by MIN-117 vs placebo at week 6. Remission with MIN-117 was achieved by 24% of patients (2.5 mg investigational product). The overall tolerability was good		Phase II, EudraCT 2015-000306-18
National Library of Medicine (2018)	MIN-117, DBRCT, *N* = 360, adult MDD patients, 6 weeks	No significant differences between active drug and placebo were detected by MADRS, HAMA, and CGI-S scores evolution		Phase II, NCT03446846
Carhart-Harris et al. (2021)	Psilocybin vs escitalopram, DBRCT, *N* = 59, moderate-to-severe MDD, 6 weeks	QIDS-SR scores at week 6 were not significantly changed vs placebo. Response rate 70% (psilocybin) vs 48% (placebo)		Phase II, NCT03429075
Griffiths et al. (2016)	Psilocybin, DBRCT, cross-over trial, *N* = 51 cancer patients + depression + anxiety, 5 weeks + 6 months follow-up	GRID-HAMD-17 and HAM-A scores were decreased by high-dose psilocybin. Quality of life, life meaning, and optimism scores improved, and death anxiety decreased under psilocybin treatment. At 6 months these changes persisted, 80% of these patients presented clinically significant decreases in depressed mood and anxiety scores		Phase II, NCT00465595
Ross et al. (2016)	Psilocybin vs niacin + psychotherapy, DBRCT, *N* = 29 patients with cancer-related anxiety and depression, 7 weeks, cross-over design	Rapid and sustained improvements in anxiety and depression before crossover, plus decreases in cancer-related demoralization and hopelessness, improvements in spiritual well-being, and quality of life. At the follow-up visit (6.5 months) consistent anxiolytic and antidepressant effects were present in the psilocybin group		Phase I, NCT00957359
Carhart-Harris et al. (2016)	Psilocybin + psychological support, open-label, *N* = 12, moderate-to-severe, treatment-resistant MDD, 3 months	The mean self-rated intensity of psilocybin effects was dose-related, and the drug was well tolerated by all patients. Depressive symptoms were markedly reduced at 1 week and 3 months compared to baseline, after high-dose treatment. Anhedonia and anxiety were markedly improved, also		Phase II, ISRCTN14426797
Davis et al. (2021)	Psilocybin, DBRCT, *N* = 24, MDD + psychotherapy, 4 weeks	The mean GRID-HAMD scores were significantly lower in the immediate treatment group, and the QIDS-SR scores reflected a rapid decrease in mean depression score after the first session, which remained significant up to week 4. In the overall sample, 71% of the participants had week 1 and week 4 clinically significant responses to the intervention. The remission rate was 58% at week 1 and 54% at week 4		Phase II, NCT03181529
COMPASS, (2021)	Psilocybin + psychological support, DBRCT, *N* = 233, treatment-resistant MDD, 4 weeks	The high dose drug (25 mg) induced a significant decrease in MADRS scores vs inactive dose after day 1, and these improvements persisted after week 3, but the difference between the low dose (10 mg) group and the control group was not significant		Phase IIb, NCT03775200
Fava et al. (2018)	Cariprazine (low doses/high doses) adjunctive to antidepressant, DBRCT, *N* = 231, treatment-resistant MDD, 19 weeks	No differences were reported on any measures between low doses of cariprazine and placebo, and higher doses led to numerically greater mean change in MADRS and CGI-I scores. MADRS response and remission rates were higher vs placebo, but without reaching statistical significance. The overall tolerability was good		Phase II, NCT00854100
Durgam et al. (2016)	Cariprazine (low doses/high doses) adjunctive to antidepressants, DBRCT, *N* = 269, treatment-resistant MDD, 8 weeks	Reductions in MADRS total score at week 8 were significantly greater for the high dose of cariprazine vs placebo, but not for the low dose. Treatment-emergent adverse events most commonly reported were akathisia, insomnia, and nausea		Phase II, NCT01469377
Earley et al. (2018)	Cariprazine adjunctive to antidepressants, DBRCT, *N* = 530, 8 weeks	Cariprazine did not significantly improve MADRS total score or SDS score vs placebo. A nonsignificant decrease in depressive symptoms was, however, recorded in the cariprazine-treated patients vs placebo group. Cariprazine improved significantly CGI-I score vs placebo, and a significantly higher proportion of patients achieved MADRS response with cariprazine vs placebo (but not significant). The overall tolerability of cariprazine was good		Phase III, NCT01715805
Fava et al., 2019	Pimavanserin as an adjunctive agent, DBRCT, *N* = 207, MDD with inadequate response to SSRI/SNRI, 10 weeks	Pimavanserin + ongoing SSRI/SNRI treatment significantly improved depressive symptoms (reflected in HAMD-17 total score change). Dry mouth, nausea, and headache were the most common adverse events in pimavanserin-treated patients. In patients with anxious depression, the response rate was 55.2 vs 22.4% (pimavanserin vs placebo) and the remission rate was 24.1 vs 5.3% (pimavanserin vs placebo), among patients with a baseline Anxiety/Somatization factor ≥7		Phase II, NCT03018340
National Library of Medicine (2019a)	Pimavanserin as adjunctive agent DBRCT, *N* = 298, MDD with inadequate response to antidepressant treatment, 5 weeks	Recruitment incomplete due to COVID-19-related problems. A 9-point HAMD total score decline at week 5 for pimavanserin treatment was reported vs 8.1 points for placebo (*p* = 0.295). A CGI-S change at week 5 of −1.4 vs −1.1 (pimavanserin vs placebo) was also reported. Response and remission rates were 31.1 and 18.2% vs 30.9 and 16.8% (pimavanserin vs placebo)		Phase III, NCT03968159
National Library of Medicine (2019b)	Pimavanserin as an adjunctive agent, *N* = 236, MDD and inadequate response to antidepressant treatment, 52 weeks	The trial was prematurely terminated “for business reasons and not due to safety concerns”		Phase III, NCT04000009
Loebel et al. (2022)	SEP-4199, DBRCT, *N* = 289/337 patients, BD type I, 6 weeks	Endpoint improvement in MADRS total score was observed on both the primary analysis (*N* = 289 participants) for SEP-4199 200 mg/day and 400 mg/day and the secondary, full ITT, analysis (*N* = 337 participants) for both regimens. Median increases in prolactin were +83.6 μg/L for the 200 mg/day dosage, +95.2 μg/L for 400 mg/day		Phase II, NCT03543410
National Library of Medicine (2021b)	SEP-4199, DBRCT, *N* = 522 (estimated), BD type I, 6 weeks	The trial is ongoing		Phase III, NCT05169710

BD, bipolar depression; CGI-I, Clinical Global Impression-Improvement; CGI-S, Clinical Global Improvement-Severity; DBRCT, double-blind randomized controlled trial; HAMA, Hamilton Anxiety Rating Scale; HAMD-17, Hamilton Depression Rating Scale; MADRS, Montgomery-Asberg Depression Rating Scale; QIDS-SR, Quick Inventory of Depressive Symptomatology - Self-rated; MDD, major depressive disorder; NLM, National Library of Medicine; SAE, severe adverse event; SDS, Sheehan Disability Scale; SNRI, Serotonin and norepinephrine reuptake inhibitor; SSRI, Selective serotonin reuptake inhibitor.

**Ansofaxine (LY03005)** is a potential triple reuptake inhibitor of serotonin, norepinephrine, and dopamine, orally administered as an extended-release tablet (Mi et al., 2021). A multicenter, randomized, double-blind, placebo-controlled, dose-finding, phase II clinical trial, conducted in China, enrolled adult MDD patients (*N* = 255) who were randomly assigned to receive fixed dose of ansofaxine (40, 80, 120, or 160 mg/day) or placebo for 6 weeks (Mi et al., 2021). Significant differences were found in the mean HAMD-17 total score changes at week 6 in all the active intervention groups vs placebo, and the overall tolerability of the drug was good (Mi et al., 2021). Treatment-related adverse events occurred in 141 patients, with an incidence of 52, 65.4, 56.8, 62.7, and 38.7% in the 40, 80, 120, 160 mg and placebo groups, respectively (Mi et al., 2021).

Another randomized, multicenter, double-blind, placebo-controlled, phase III study evaluated the efficacy and safety of ansofaxine hydrochloride extended-release tablets in 558 Chinese adult patients diagnosed with MDD (National Library of Medicine [NLM], NCT04853407). According to the manufacturer’s site, the results of this trial showed that LY03005 (80 mg or 160 mg/day) was safe and effective at week 8, with statistically significant improvements in both primary (Montgomery Asberg Depression Rating Scale- MADRS total score) and secondary (Hamilton Depression Rating Scale-HAMD-17, Clinical Global Impression- CGI, Hamilton Anxiety Rating Scale- HAM-A, HAMD-17 Anxiety/Somatization factor, HAMD-17 Cognitive Impairment Factor, HAMD-17 Blocking factor, MADRS Anhedonia Factor Score and Sheehan Disability Scale total score) endpoints vs placebo (Luye Pharma, 2021). No serious adverse events occurred during this trial, and common adverse events (>5% incidence) in the LY03005 group were nausea, vomiting, headache, and drowsiness (NLM, NCT04853407).

**Edivoxetine (LY2216684)** is a highly selective norepinephrine reuptake inhibitor investigated for the treatment of MDD, as an adjunctive agent to the current antidepressant (Ball et al., 2016). Analysis of data derived from three randomized, phase III, 8-week, placebo-controlled trials, with a 3-week double-blind placebo lead-in phase, that evaluated the efficacy of edivoxetine (6–18 mg/day) as an adjunctive treatment for patients with MDD and partial response to SSRIs did not support a significant improvement in the clinical status of these patients (701, 689, and 449 participants) (Ball et al., 2016). The primary outcome was the mean change from baseline to week 8 in the MADRS total score, but each trial failed to meet this primary objective and most of the secondary objectives (Ball et al., 2016).

A phase III trial included an 8-week, open-label phase that evaluated edivoxetine (12–18 mg/day, flexible-dose regimen) as adjunctive to selective serotonin reuptake inhibitors (SSRI) treatment in 1249 MDD patients (Oakes et al., 2015). This first phase was followed by a 12-week open-label stabilization if participants were in remission at the end of week 8, followed by a randomized, double-blind, placebo-controlled period of 24 weeks (Oakes et al., 2015). In the last phase of the trial, 294 patients were randomized to continue adjunctive edivoxetine, and 292 were switched to adjunctive placebo (Oakes et al., 2015). The comparison of the two groups at the end of the study, based on the MADRS total score change, did not show the presence of significant differences in time to re-emergence of symptoms, rates of symptom re-emergence, or rates of sustained remission (Oakes et al., 2015).

A phase II, double-blind, placebo-controlled, 10-week therapy of adjunctive edivoxetine (6–18 mg/day) or adjunctive placebo with SSRI, which enrolled 131 participants, did not report a significant difference in the primary outcome change from baseline to week 8 in the MADRS total score, with a 0.26 effect size (Ball et al., 2014). Significant treatment differences favoring edivoxetine were shown only in the role functioning and the functional impact of the fatigue (Ball et al., 2014).

In another randomized, double-blind, placebo-controlled trial, LY2216684 (6–18 mg/day) was evaluated in 495 adult MDD patients for 10 weeks (Pangallo et al., 2011). The investigational product improved significantly MADRS scores vs placebo from baseline to week 10, and it was also associated with a higher probability of achieving response (49.5%) and remission (29.7%) compared with placebo (29.3 and 18.8%, respectively) (Pangallo et al., 2011). For Sheehan Disability Scale (SDS) global functional impairment score, LY2216684 administration resulted in significantly greater improvement compared with placebo, and more edivoxetine-treated patients discontinued the study due to adverse events or death (Pangallo et al., 2011).

A multicenter, 54-week, open-label trial of adjunctive edivoxetine 12 or 18 mg once daily in MDD patients with partial response to the current SSRI therapy (*N* = 328 participants completed the trial) showed mean improvements of −17 points on MADRS from baseline to week 54, and a rate of study discontinuation due to adverse events of 17% (Ball et al., 2015). Treatment-emergent adverse events most commonly reported were nausea, hyperhidrosis, constipation, headache, dry mouth, dizziness, vomiting, insomnia, and upper respiratory tract infection (Ball et al., 2015).

**MIN-117** is a potential antidepressant agent with α1A, α1B, 5HT1A, and 5HT2A receptors modulator properties; this product also possesses serotonin and dopamine transporter reuptake inhibition activity (Davidson et al., 2016). A four-arm, parallel-group, multicentric, randomized, double-blind, placebo- and positive-controlled trial evaluated two doses (0.5 and 2.5 mg) of MIN-117 in 84 patients with moderate-to-severe MDD, to detect a signal and to estimate the effect size (Davidson et al., 2016). A dose-dependent superiority of MIN-117 over placebo, determined by MADRS scores change at week 6, was demonstrated (Davidson et al., 2016). The effect size for the 2.5 mg dose of MIN-117 was 0.33 compared with placebo, and 0.23 for the lower dose (Davidson et al., 2016). Remission was achieved by 24% of the patients treated with 2.5 mg MIN-117, and both doses of the investigational product were well tolerated, without differences in the incidence and types of adverse events between MIN-117 and placebo (Davidson et al., 2016). Another randomized, double-blind, parallel-group, placebo-controlled phase II study investigated the efficacy and safety of MIN-117 in 360 adult patients diagnosed with MDD, monitored for 6 weeks (NLM, NCT03446846). The results posted on clinicaltrials.gov did not support the existence of significant differences between active drug and placebo in either the primary outcome (MADRS score change) or secondary outcomes (HAMA and CGI-S score changes) for any of the tested doses (NLM, NCT03446846).

The psychedelic molecule **psilocybin** (4-phosphoryloxy-N,N-dimethyltryptamine) has been associated with positive results in clinical trials dedicated to depression and anxiety treatment (Carhart-Harris et al., 2021). This compound has 5HT2A receptor antagonism, a pathway exploited by other products with antidepressant properties, e.g. trazodone, nefazodone, or mirtazapine (Celada et al., 2004; Carhart-Harris et al., 2021). In a phase II, double-blind, randomized, controlled trial, patients with a long history of moderate-to-severe depression (*N* = 59) received either psilocybin or escitalopram, over 6 weeks of treatment (Carhart-Harris et al., 2021). Psilocybin was administered as 25 mg doses separated by 3 weeks, plus 6 weeks of daily placebo, or as two distinct doses of 1 mg 3 weeks apart plus 6 weeks of daily oral escitalopram treatment (Carhart-Harris et al., 2021). After 6 weeks, the difference between groups in terms of QIDS-SR (Quick Inventory of Depressive Symptomatology- Self-reported) scores was not significant (*p* = 0.17), with a response being detected in 70% of the patients treated with psilocybin+placebo vs 48% in the group receiving psilocybin+escitalopram treatment (Carhart-Harris et al., 2021). The remission rates, based also on QIDS-SR scores, were 57 and 28%, respectively, while other secondary outcomes generally favored psilocybin vs escitalopram, and the incidence of adverse events was similar in both trial groups (Carhart-Harris et al., 2021). In conclusion, this trial did not support the efficacy of psilocybin in comparison with escitalopram, at least in the main outcome treatment (Carhart-Harris et al., 2021).

A randomized, double-blind, cross-over trial investigated the effects of a very low dose (1 or 3 mg/70 kg)-equivalent to placebo vs a high dose (22 or 30 mg/70 kg) of psilocybin administered in a counterbalanced sequence with 5 weeks between sessions and a 6-month follow-up, in 51 cancer patients with life-threatening diagnoses and symptoms of depression and/or anxiety treatment (Griffiths et al., 2016). The two primary outcome measures were clinician-rated GRID-HAMD-17 and HAM-A scores (Griffiths et al., 2016). Other scales evaluated general psychiatric symptoms, quality of life, self-rated optimism concerning own illness, anxiety about death, attitudes toward death, and life meaningfulness (Griffiths et al., 2016). The subjective effect measures were assessed 7 h after psilocybin, using the Hallucinogen Rating Scale, Mysticism Scale, 5-Dimension Altered States of Consciousness, and the States of Consciousness Questionnaire (Griffiths et al., 2016). High-dose psilocybin decreased clinician- and self-rated measures of depressed mood and anxiety, increased quality of life, life meaning, and optimism scores, and also decreased death anxiety (Griffiths et al., 2016). At 6-month follow-up, these changes persisted, as 80% of the patients continued to show clinically significant decreases in depressed mood and anxiety (Griffiths et al., 2016).

In a double-blind, 7-week, cross-over, placebo-controlled trial, 29 patients with cancer-related anxiety and depression were randomized to treatment with single-dose psilocybin (0.3 mg/kg) or niacin, both in conjunction with psychotherapy (Ross et al., 2016). Before the crossover, psilocybin induced rapid and sustained improvements in anxiety and depression and led to decreases in cancer-related demoralization and hopelessness, improved spiritual well-being, and quality of life (Ross et al., 2016). At the follow-up visit (6.5 months), psilocybin was associated with consistent anxiolytic and antidepressant effects (60–80% of the participants still benefitted from the intervention), sustained existential distress reductions, quality of life amelioration, and improvements in attitude toward death (Ross et al., 2016). The psilocybin-induced mystical experience is assumed to have mediated the therapeutic effect of psilocybin on mood and anxiety symptoms (Ross et al., 2016).

An open-label, feasibility trial enrolled 12 patients with moderate-to-severe, unipolar, treatment-resistant major depression, who received two oral doses of psilocybin (10 and 25 mg, 7 days apart) in conjunction with psychological support (Carhart-Harris et al., 2016). The mean self-rated intensity of psilocybin effects was dose-related, and the drug was well tolerated by all the patients (Carhart-Harris et al., 2016). The adverse reactions were transient anxiety during drug onset, transient confusion or thought disorder, mild and transient nausea, and transient headache (Carhart-Harris et al., 2016). Depressive symptoms were markedly reduced at 1 week and 3 months, compared with baseline, after high-dose treatment (Carhart-Harris et al., 2016). Anhedonia and anxiety were markedly improved and these changes were maintained for long periods (Carhart-Harris et al., 2016).

In a randomized clinical trial, 24 participants with MDD who received immediate psilocybin-assisted therapy or delayed treatment were compared using clinician-rated assessments of depression severity (GRID-HAMD-17) and self-reported (QIDS-SR) for 1 month (Davis et al., 2021). Two psilocybin sessions (20 mg/70 kg—first session, 30 mg/70 kg—second session) were given, in the context of supportive psychotherapy (approximately 11 h), and patients were randomized to begin treatment immediately or after an 8-week delay (Davis et al., 2021). The mean GRID-HAMD scores were significantly lower in the immediate treatment group, and the QIDS-SR scores reflected a rapid decrease in the mean depression score after the first session, which remained significant up to week 4 (Davis et al., 2021). In the overall sample, 71% of the participants had at week 1 and week 4 a clinically significant response to the intervention (≥50% reduction in GRID-HAMD score) (Davis et al., 2021). The remission rate was 58% at week 1 and 54% at week 4 (score ≤7 on GRID-HAMD) (Davis et al., 2021).

In a randomized, multicenter, controlled, double-blind, phase IIb clinical trial, a single dose of COMP360 (psilocybin) was given to 233 patients with treatment-resistant depression, in conjunction with psychological support (COMPASS, 2021). All patients discontinued antidepressants before participation in this trial (COMPASS, 2021). Psilocybin was administered as 10 mg, 25 mg, or a comparator dose of 1 mg (COMPASS, 2021). The high-dose drug led to a significant decrease in MADRS scores vs inactive dose after day 1, and these improvements persisted after week 3, but the difference between the low-dose (10 mg) group and the control group was not significant (COMPASS, 2021). At least twice the number of patients in the high-dose group showed response and remission at week 3 and week 12, compared with the 1-mg group (COMPASS, 2021). The overall tolerability of COMP360 was good, with more than 90% of treatment-emergent adverse events being mild or moderate in intensity (COMPASS, 2021).

**Cariprazine** is an atypical antipsychotic under investigation as an adjuvant agent to antidepressants in patients diagnosed with MDD. Cariprazine is an orally active agent that possesses a 10-fold higher affinity for dopamine D3 receptors than for D2 receptors (partial agonist) and potent serotonin 5HT1A receptor partial agonist properties; its active metabolite, didesmethyl-cariprazine, has a half-life of 1–3 weeks (Earley et al., 2018; Citrome, 2019). This antipsychotic is FDA-approved for the treatment of schizophrenia, manic/mixed episodes in bipolar I disorder, and bipolar depression (Citrome, 2019; Vasiliu, 2021). Its antidepressive potential is attributed to the high affinity for and occupancy of D3 receptors, which are localized in motivation and reward-related brain areas (Earley et al., 2018). Based on the analysis of pivotal registration trials with cariprazine in bipolar depression (*n* = 4 studies), rates of treatment response (≥50% reduction of MADRS total score at endpoint) under cariprazine 1.5–3 mg/day treatment vs placebo were 46.3 vs 35.9% (NNT = 10) (Citrome, 2019). Based on the same analysis, the rates for remission (≤10 MADRS final total score) were 30.2 vs 20.9% (cariprazine vs placebo), leading to an NNT value of 11. The discontinuation rates due to adverse events were 6.7% for cariprazine vs 4.8% for placebo (NNH = 51) (Citrome, 2019).

A double-blind, placebo-controlled, randomized, 19-week phase II study evaluated the efficacy, safety, and tolerability of adjunctive cariprazine (0.1–0.3 mg and 1.0–2.0 mg/day) as an antidepressant treatment for adults with treatment-resistant MDD (*N* = 231) (Fava et al., 2018). No differences were reported on any measures between low doses of cariprazine and placebo. Higher doses led to numerically greater mean change in MADRS and CGI-I scores, and MADRS response and remission rates vs placebo, but without reaching statistical significance (Fava et al., 2018). The overall tolerability was good and adverse events in both dosage groups included headache, arthralgia, restlessness, fatigue, increased appetite, insomnia, dry mouth, and constipation (Fava et al., 2018). Another randomized, double-blind, placebo-controlled, flexible-dose study evaluated adult patients diagnosed with MDD with an inadequate antidepressant response, who were randomized to 8-week adjunctive treatment with placebo (*N* = 269), cariprazine 1–2 mg/day (*N* = 274), or cariprazine 2–4.5 mg/day (*N* = 276) (Durgam et al., 2016). Reductions in MADRS total score at week 8 were significantly greater for the high dose of cariprazine vs placebo, but not for the low dose (Durgam et al., 2016). The favorable effect was detected early, at weeks 2, 4, and 6 for the 2–4.5 mg/day regimen, and at weeks 2 and 4 for the 1–2 mg/day regimen (Durgam et al., 2016). Treatment-emergent adverse events most commonly reported were akathisia, insomnia, and nausea (Durgam et al., 2016).

The results of a double-blind, randomized, placebo-controlled, phase III study evaluating the efficacy of adjunctive cariprazine (1.5–4.5 mg/day) added to tricyclics in patients with previous inadequate response to monotherapy with antidepressants (*N* = 530 participants) showed that cariprazine did not significantly improve MADRS total score or SDS score vs placebo (Earley et al., 2018). A nonsignificant decrease in depressive symptoms was, however, recorded in the cariprazine-treated patients vs the placebo group (Earley et al., 2018). Cariprazine improved significantly CGI-I score vs placebo, and a significantly higher proportion of patients achieved MADRS response with cariprazine vs placebo (but not significant) (Earley et al., 2018). The overall tolerability of cariprazine was good, with metabolic parameters and body weight changes not being different from placebo (Earley et al., 2018). Akathisia and restlessness were the most commonly reported adverse events (Earley et al., 2018).

**Pimavanserin** is an approved drug for the treatment of Parkinson’s disease psychosis, and it possesses potent 5HT2A receptor antagonist/inverse agonist properties, with lesser activity as a 5HT2C antagonist/inverse agonist, and no interaction with adrenergic, dopaminergic, histaminergic, or muscarinic receptors (Fava et al., 2019). The results of a multicenter, randomized, double-blind, placebo-controlled, phase II study (the CLARITY trial) in patients with MDD and inadequate response to an SSRI or serotonin and norepinephrine reuptake inhibitor (SNRI) (*N* = 207 participants) showed that the addition of pimavanserin to ongoing SSRI/SNRI treatment may lead to significant improvements in depressive symptoms (reflected in HAMD-17 total score change, *p* = 0.004) (Fava et al., 2019). Early and sustained separation of pimavanserin from placebo at a statistically significant level occurred at week 1 visit (Fava et al., 2019). Dry mouth, nausea, and headache were the most common adverse events in pimavanserin-treated patients (Fava et al., 2019).

The effect of pimavanserin on anxious depression was determined from the data collected in the CLARITY trial, and the anxiety/somatization (AS) factor, derived from the HAMD items, decreased significantly in patients with initially high AS scores (≥7) (Papakostas et al., 2020). The response rate (≥50% reduction in HAMD-17 from baseline) was 55.2 vs 22.4% (pimavanserin vs placebo) and the remission rate (HAMD final score <7) was 24.1 vs 5.3% (pimavanserin vs placebo), among patients with a baseline AS factor ≥7 (Papakostas et al., 2020). Therefore, adjunctive pimavanserin to the current antidepressant treatment seems efficient in patients with anxious MDD (Papakostas et al., 2020).

A phase III, randomized, multicenter, double-blind, placebo-controlled study enrolled 298 participants diagnosed with MDD and inadequate response to antidepressant treatment to evaluate the efficacy and safety of adjunctive pimavanserin 34 mg/day, with the main outcome measure being the change from baseline to week 5 in HAMD-17 total score (NLM, NCT03968159). The recruitment was halted by the sponsor due to the COVID outbreak, but the data posted on clinicaltrials.gov show a nine-point HAMD total score decline at week 5 for pimavanserin treatment and 8.1 for placebo (*p* = 0.295), and a CGI-S change at week 5 of −1.4 vs. −1.1 (pimavanserin vs placebo) (NLM, NCT03968159). The response and remission rates were 31.1 and 18.2% vs 30.9 and 16.8% (pimavanserin vs placebo) (NLM, NCT03968159). Therefore, no significant difference seems to exist between active treatment and placebo in the main variables. The rate of serious adverse events was similar in the two groups (NLM, NCT03968159).

A phase III, 52-week extension study to assess the safety and tolerability of adjunctive pimavanserin in patients with MDD and inadequate response to antidepressant treatment was prematurely terminated “for business reasons and not due to safety concerns,” according to the sponsor’s announcement posted on clinicaltrials.gov (NLM, NCT04000009). No results have been made publicly available as of February 2022.

**Atypical antipsychotics with antagonist activity at the** serotonin **5-HT7 receptors have been associated with antidepressant efficacy** (Loebel et al., 2022). SEP-4199 is a non-racemic amisulpride represented by an 85:15 ratio of R-amisulpride:S-amisulpride (Loebel et al., 2022). The investigational oral drug is described pharmacodynamically as possessing increased potency for 5-HT7 receptors vs dopamine D2 receptors (because of different affinity for these receptors by enantiomers), which is expected to be beneficial for the treatment of bipolar depression (Loebel et al., 2022). In a randomized, 6-week, double-blind, placebo-controlled trial, endpoint improvement in the MADRS total score was observed on both the primary analysis (*N* = 289 participants) for SEP-4199 200 mg/day and 400 mg/day, and on the secondary, full ITT, analysis (*N* = 337 participants) for both regimens, in patients with bipolar I depression (Loebel et al., 2022). Low rates of individual adverse events were reported (under 8%) and minimal effects on weight and lipids were detected. Median increases in prolactin were +83.6 μg/L for the 200 mg/day dosage, +95.2 μg/L for 400 mg/day vs no increase on placebo (Loebel et al., 2022). Another phase 3, randomized, double-blind, placebo-controlled, parallel-group study is ongoing, and it has as objective the evaluation of the efficacy, safety, and tolerability of SEP-4199 CR at a fixed dose of 200 mg/day or 400 mg/day in patients diagnosed with bipolar I depression, during 6 weeks, with an estimated enrollment of 522 participants (NLM, NCT05169710).

In conclusion, antidepressants targeting the monoaminergic system are still actively researched, although the available data are mixed. Ansofaxine, MIN-117, cariprazine, pimavanserin, and SEP-4199 are the most promising molecules in this category. Also, it should be mentioned that many investigational products within this class have been discontinued from clinical research (Perez-Caballero et al., 2019). Therefore, the attention of the researchers has been focused on a different, non-monoaminergic mechanism for future antidepressants.

## Orexin Receptors Antagonists

Orexins (hypocretins) are produced from the same precursor peptide, prepro-orexin, in the lateral and posterior hypothalamus (Han et al., 2020). Type A and type B orexins (or hypocretin-1 and -2) are ligands for type 1 and type 2 receptors (OX1R,2R) which are protein G-coupled, and modulate functions such as feeding, sleep, and motivated behaviors (Sakurai, 2014; Han et al., 2020). OX1R have a higher affinity for orexin-A and OX2R present a similar affinity for both orexin-A and B (Jha, 2021). Selective OX1R antagonists (SORA1) may be useful in the treatment of anxiety and drug addiction, selective OX2R antagonists (SORA2) are investigated in animal models for the therapy of sleep disorders, while dual OX1R and OX2R antagonists (DORA) are already marketed for the treatment of insomnia (Staton et al., 2018; Han et al., 2020). **Seltorexant (MIN-202, JNJ-42847922, JNJ-922)** is a SORA2 agent studied for the treatment of insomnia and MDD (Recourt et al., 2019). This agent may normalize excessive arousal and attenuate depressive symptoms, and in a randomized, double-blind, diphenhydramine-, and placebo-controlled study (*N* = 47 MDD patients) it significantly improved after 10 days’ core depressive symptoms compared with placebo (Recourt et al., 2019). The antidepressant efficacy of seltorexant was maintained with continued treatment for up to 28 days, and this effect coincided with a relative increase in delta- and decreased theta-, alpha-, and beta power during stage 2 sleep (Recourt et al., 2019).

In a phase IIb, randomized, placebo-controlled, adaptive dose-finding study, 287 patients with MDD who had an insufficient response to 1-3 SSRI/SNRI during the current episode were randomized to placebo or seltorexant (20 or 40 mg) as add-ons to the currently administered antidepressant (Savitz et al., 2021). A significant reduction in depressive symptoms (MADRS scores) was observed for seltorexant (20 mg), and in the subset of patients with sleep disturbance, the difference between seltorexant 20 mg and placebo was larger (Savitz et al., 2021).

Another phase I, randomized, 6-week, double-blind, placebo-controlled trial is currently exploring the efficacy of seltorexant as adjunctive therapy to antidepressants in adolescents with MDD who have an inadequate response to SSRIs and psychotherapy (NLM, NCT04951609). The estimated enrollment is reported to be 52 patients, and the outcomes will be related to tolerability, depression severity, clinical global impression, sleep quality, cognitive performance, and pharmacokinetic parameters (NLM, NCT04951609).

A synthesis of the data regarding the clinical trials focused on the orexinergic modulators with antidepressant properties in the pipeline is presented in Table 4. Based on the reviewed data, seltorexant may be useful as monotherapy for MDD, and as an add-on agent for treatment-resistant depression, but more trials with a longer duration of monitoring are needed.

**TABLE 4:** Orexinergic agents with antidepressant properties in the pipeline.

Authors/Trial sponsor	Methodology	Results	Clinical trial phase, trial identifier (if available)
Recourt et al. (2019)	Seltorexant (MIN-202, JNJ-42847922, JNJ-922) vs diphenhydramine vs placebo, DBRCT, *N* = 47, MDD, 4 weeks	Core symptoms of depression were improved after 10 days with seltorexant vs placebo and its efficacy persisted up to day 28	Phase Ib, NCT02476058
Savitz et al. (2021)	Seltorexant + ongoing antidepressant, DBRCT, *N* = 287, MDD with insufficient response to 1–3 SSRI/SNRI, 6 weeks	MADRS scores improved more in the seltorexant (20 mg) vs placebo at weeks 3 and 6. If baseline ISI≥15 the efficacy of seltorexant 20 mg/day was higher vs placebo	Phase IIb, NCT03227224
National Library of Medicine (2021c)	Seltorexant + ongoing antidepressant, DBRCT, *N* = 52 (estimated), MDD with inadequate response to SSRI/psychotherapy	The outcomes will be related to tolerability, depression severity, clinical global impression, sleep quality, cognitive performance, and pharmacokinetic parameters	Phase I, NCT04951609

DBRCT, double-blind randomized controlled trial; ISI, Insomnia Severity Index; MADRS, Montgomery-Asberg Depression Rating Scale; MDD, major depressive disorder; NLM, National Library of Medicine; SNRI, Serotonin and norepinephrine reuptake inhibitor; SSRI, Selective serotonin reuptake inhibitor.

## GABA-A Receptor Modulators and Neurosteroid Analogs

One of the extensively explored pathophysiological mechanisms for MDD is the dysfunction of the GABA neurotransmission and the downregulation of neurosteroid biosynthesis (Schüle et al., 2014; Hoffmann et al., 2020). It is presumed that alteration of the transmembrane channels that make up GABA-A receptors can induce anxiety and neurodevelopmental disorders (Edinoff et al., 2021; Zhu et al., 2018). Reduced levels of allopregnanolone in the peripheral circulation or cerebrospinal fluid were associated not only with major depression, anxiety disorders, premenstrual dysphoric disorder, but also with negative symptoms of schizophrenia or impulsive aggression (Schüle et al., 2014). Based on the observation that allopregnanolone, an endogenous neuroactive steroid that possesses GABA-A receptor-positive allosteric modulating properties, may improve symptoms of depression and anxiety by intensifying GABA-ergic signaling in the central nervous system (Schüle et al., 2014); a series of these steroid analogs have been tested for the treatment of MDD. Besides the role of GABA-ergic neurotransmission regulation, allopregnanolone may have positive effects on mood disorders by the enhancement of neurogenesis, myelination, neuroprotection, and regulatory effects on the hypothalamus-pituitary-adrenocortical (HPA) axis (Schüle et al., 2014).

**Brexanolone (SAGE-547)** is the first FDA-approved intravenous treatment for postpartum depression and represents a soluble, β-cyclodextrin-based form of the neuroactive steroid allopregnanolone (Cooper et al., 2019). Due to its binding to GABA-A receptors, brexanolone enhances the inhibitory effects of GABA when it occupies these receptors, leading to an acute decrease in anxiety levels and depression symptoms (Schüle et al., 2014). Brexanolone is not recommended for patients engaging in activities that require high levels of alertness, and it should be avoided in end-stage renal disease (eGFR<15 ml/min/m^2^) (Edinoff et al., 2021).

In a proof-of-concept, phase II, label study of brexanolone, which included four women with severe post-partum depression (HAMD≥20), titrated to a dose similar to third-trimester allopregnanolone levels, this antidepressant was associated with 14 adverse events, but none of them was severe (Kanes SJ. et al., 2017). The mean HAMD score decreased to levels suggesting remission of symptoms, after 84 h of monitoring (Kanes SJ. et al., 2017).

In a phase II trial, brexanolone decreased at 60 h the total HAMD score with a significantly greater impact than placebo (Kanes S. et al., 2017). The overall tolerability of brexanolone was good, with no serious adverse events or discontinuations due to adverse events (Kanes S. et al., 2017). The most frequently reported adverse effects were dizziness, somnolence, and sinus tachycardia (Kanes S. et al., 2017).

In two double-blind, multicentric, phase III trials, women with post-partum depression (N_1_ = 138, and N_2_ = 108, respectively), of severe intensity (HAMD≥26 for one trial, and 20–25 for the other trial) received a single i.v. injection of brexanolone 90 or 60 μg/kg vs placebo for 60 h (first trial), or brexanolone 90 μg/kg vs placebo (trial 2) for the same duration (Meltzer-Brody et al., 2018). Patients who received brexanolone in both trials presented significant clinical improvement, according to the HAMD scores, after 60 h vs placebo, with rapid onset of the therapeutic action and long-lasting treatment response (evaluated up to 30 days) (Meltzer-Brody et al., 2018). The most frequently reported adverse events in the brexanolone groups were headache, dizziness, and somnolence (Meltzer-Brody et al., 2018).

A *posthoc* analysis of three trials conducted with brexanolone (*N* = 299 women with post-partum depression) showed a superior effect for the active drug vs placebo after 60 h and at day 30 (Meltzer-Gerbasi et al., 2021). Significantly more patients treated with brexanolone than those who received placebo achieved minimal, moderate, and large HAMD-17 score change at hour 60, as well as a large meaningful response at day 30 (Meltzer-Gerbasi et al., 2021). Also, patients treated with brexanolone had a higher probability to sustain HAMD-17 remission and CGI-I response until day 30 vs the placebo-treated group (Meltzer-Gerbasi et al., 2021).

A review that extracted data from 26 studies dedicated to pharmacological and pharmacological/nonpharmacological combination therapies in postpartum depression concluded that matching-adjusted indirect comparisons between brexanolone and placebo arms of comparator studies, on one side, and between SSRIs vs placebo, on the other side, lead to larger differences in the change from baseline scores in HAMD and Edinburgh Postnatal Depression Scale (EPDS) in favor of brexanolone vs SSRIs (Cooper et al., 2019). The differences in HAMD scores were between 12.79 (day 3) and 0.97 (last observation), whereas the EPDS score difference varied between 7.98 (day 3) and 4.05 (last observation) (Cooper et al., 2019).

Zuranolone (SAGE-217) **is a neuroactive steroid and GABA-A receptor-positive allosteric modulator that shares a similar pharmacodynamic profile as brexanolone injection** (Martinez Botella et al., 2017). Both zuranolone and brexanolone have an affinity for synaptic (γ subunit-containing) as well as for extrasynaptic (δ subunit-containing) GABA-A receptors, but the first agent has been formulated for oral administration and once-daily dosing (Althaus et al., 2020). In preclinical models, zuranolone potentiated GABA currents synergistically with diazepam, in a noncompetitive manner (Althaus et al., 2020).

In two phase I studies of SAGE-217, which included 108 healthy volunteers, the investigational product was well tolerated and its pharmacokinetic profile was well characterized (Hoffmann et al., 2020). In a double-blind, phase II trial, 89 patients with MDD were randomized on 30 mg SAGE-217 or placebo once daily, and they were monitored using change in the HAMD scores as the main outcome (Gunduz-Bruce et al., 2019). Administration of SAGE-217 for 14 days resulted in significant improvements in depressive symptoms compared with placebo (Gunduz-Bruce et al., 2019). There were reported no serious adverse events, and the most common adverse events in the active substance group were headache, dizziness, nausea, and somnolence (Gunduz-Bruce et al., 2019).

In a phase III, double-blind, randomized, outpatient, placebo-controlled trial, 153 patients diagnosed with post-partum depression were assigned to treatment with zuranolone (30 mg) or placebo for 14 days and were monitored using HAMD-17 scores as the primary outcome (Deligiannidis et al., 2021). On day 15, zuranolone improved HAMD scores from baseline vs placebo, and this trend to superiority was observed from day 3 and persisted until day 45 (end of study visit) (Deligiannidis et al., 2021). Significant differences between zuranolone and placebo were observed in the therapeutic response rate, remission rate, and MADRS score improvement, whereas HAMA scores also improved significantly (Deligiannidis et al., 2021). The treatment was generally well tolerated; one patient experienced a serious adverse event (confusional state) and one was discontinued because of an adverse event (Deligiannidis et al., 2021).

A phase III trial evaluating the efficacy of SAGE-217 in adults with severe post-partum depression is ongoing, with an estimated completion date of September 2022 (NLM, NCT04442503). A number of 192 patients are expected to be enrolled in this trial and monitored for 14 days, with the primary outcome being the severity of depression, determined by the HAMD-17 score on day 15 (NLM, NCT04442503).

**Ganaxolone (CCD1042)** is an allopregnanolone analog explored mainly as an anticonvulsant, but also as an adjunctive agent for the treatment of persistent depression in postmenopausal women and as monotherapy in postpartum depression (Dichtel et al., 2020).

In an open-label clinical trial (*N* = 10 participants, mean age 62.8 ± 6.3 years) ganaxolone (225 mg b.i.d., increased to 450 mg b.i.d. if tolerated) was administered orally for 8 weeks in cases where an adequately dosed antidepressant did not lead to response after ≥6 weeks (Dichtel et al., 2020). Ganaxolone was associated with a favorable evolution, with MADRS scores decreasing after 8 weeks, and this decrease persisted over a 2-week taper, with 44% of the subjects who completed the 8-week treatment period experiencing response (MADRS score decrease ≥50%) and remission (final MADRS<10) (Dichtel et al., 2020). The response and remission rates persisted in 100 and 50% of subjects at 10 weeks, and the secondary endpoints showed also significant improvement (sleep quality, changes in appetite, and weight) (Dichtel et al., 2020). Sleepiness, fatigue, and dizziness were reported as adverse events during this trial, without significant effects on quality of life or sexual function (Dichtel et al., 2020).

In a phase 2 trial, ganaxolone was administered i.v. at median doses of 60, 90, and 140 μg/kg/h as a 60-h infusion in patients with severe postpartum depression (*N* = 58, HAMD≥26) (Marinus Pharmaceuticals, 2018). Ganaxolone was efficient, with the most robust results being reported in the highest dose group. A clinically meaningful reduction in the HAMD-17 total score vs placebo was reported at 48 h and this improvement was maintained until the last visit, on day 34 (Marinus Pharmaceuticals, 2018). The rate of response was high on day 34 and after 60 h (75%, and 67%, respectively), and also the rate of remission was important (54 and 33%, respectively) (Marinus Pharmaceuticals, 2018). Sedation and dizziness were the most frequently reported adverse events, but no serious adverse event/discontinuation due to adverse events was observed (Marinus Pharmaceuticals, 2018). In the second part of this trial, 33 patients with postpartum depression received a 6-h infusion with ganaxolone (20 mg/h), and then oral ganaxolone 900 mg daily or placebo for 28 days (Marinus Pharmaceuticals, 2019). The HAMD-17 scores decreased rapidly at 6 h, but on day 28 there was no significant difference between the active drug and placebo (Marinus Pharmaceuticals, 2019). Sedation, dizziness, and somnolence lasted between 2 and 10 days, except for one case where sedation lasted throughout the treatment period (Marinus Pharmaceuticals, 2019). The secondary outcomes (CGI, EPDS, and Spielberger State-Trait Anxiety 6- STAI-6) showed similar trends with the HAMD-17 scores (Marinus Pharmaceuticals, 2019).

In another, open-label study, 25 patients diagnosed with postpartum depression received 675 mg of oral ganaxolone for 28 days, and 43 patients received 675 mg of oral ganaxolone for 2 days, followed by 1125 mg once daily for the remainder of the study (Marinus Pharmaceuticals, 2019). The high-dose regimen was superior as reflected by the evolution of the HAMD-17 scores, and this trend was maintained over the treatment regimen (Marinus Pharmaceuticals, 2019). The onset of the favorable effect was detected at 24 h, and the treatment was generally safe and well-tolerated, with no serious adverse events/discontinuation due to adverse events being reported (Marinus Pharmaceuticals, 2019).

**PRAX-114** is a GABA-A receptor-positive allosteric modulator that achieves 10.5-fold greater potentiation of extrasynaptic receptors vs synaptic receptors in animal models (Hughes et al., 2021). Two clinical trials dedicated to the efficacy and tolerability of oral PRAX-114 in MDD are ongoing: the first one is a phase II/III trial, randomized, double-blind, placebo-controlled, which will compare the effects of 40 mg active drug in 200 participants, using HAMD as a primary outcome measure; the second trial will evaluate the effects of PRAX-114 as adjunctive treatment (10, 20, 40, or 60 mg/day) vs placebo over HAMD scores in a phase II, randomized, double-blind design, with an expected enrollment of 125 participants with MDD and inadequate response to antidepressant treatment (NLM, NCT04832425; NLM, NCT04969510).

In conclusion, neurosteroid analogs are interesting therapeutic options for the treatment of depressive disorders (postpartum depression, MDD, postmenopausal depression, treatment-resistant depression), with brexanolone being already marketed for post-partum depression. It is expected that the inconvenience of i.v. administration in the case of brexanolone is to be overcome by the other pregnanolone analogs (Table 5).

**TABLE 5:** Neurosteroid analogs and GABA-A receptor modulators with antidepressant properties in the pipeline.

Authors/Trial sponsor	Methodology	Results	Clinical trial phase, trial identifier (if available)
Kanes et al. (2017a)	Brexanolone (SAGE-547), open-label, *N* = 4, PPD, 84 h	Mean HAMD and CGI-I scores had favorable evolution; 14 adverse events were reported, but no SAE	Phase II, NCT02285504
Kanes et al. (2017b)	Brexanolone, DBRCT, *N* = 21, severe PPD, 60 h	HAMD total scores decreased significantly vs placebo at 60 h. Dizziness and somnolence were the most frequently reported adverse events	Phase II, NCT02614547
Meltzer-Brody et al. (2018)	Brexanolone, two DBRCT, *N* = 138 and 108, severe PPD, 60 h	HAMD scores evolution supported the existence of a significant clinical improvement vs placebo, which persisted up to 30 days. Headache, dizziness, somnolence were the most commonly reported adverse events	Phase III, NCT02942004
			Phase III, NCT02942017
Gerbasi et al. (2021)	Brexanolone, post-hoc analysis of three trials, *N* = 299, PPD, 30 days	Brexanolone was superior to placebo after 60 h and 30 days. Higher probability to sustain HAMD-defined remission and CGI-I response vs placebo at day 30	Phase II, NCT02614547
			Phase III, NCT02942004
			Phase III, NCT02942017
Hoffmann et al. (2020)	Zuranolone (SAGE-217), two trials, DBRCT, *N* = 108 healthy volunteers (72 and 36, respectively), single ascending dose study and multiple ascending dose study	Safety, tolerability, and pharmacokinetics of SAGE-217. Mild and transient sedation was observed. Most adverse events were reported as mild/moderate intensity. No SAE was reported	Phase I
Gunduz-Bruce et al. (2019)	Zuranolone, DBRCT, *N* = 89, MDD, 14 days	HAMD scores improved significantly vs placebo, no SAE was reported. Dizziness, headache, nausea, and somnolence were the most common adverse events	Phase II, NCT03000530
Deligiannidis et al. (2021)	Zuranolone, DBRCT, *N* = 153, PPD, 45 days	HAMD scores were improved by zuranolone vs placebo from day 3, up to day 45. HAMA and MADRS also improved under zuranolone treatment vs placebo. The overall tolerability of zuranolone was good, with one SAE (confusional state)	Phase III, NCT02978326
National Library of Medicine (2020)	Zuranolone, DBRCT, *N* = 192, severe PPD, 14 days	HAMD-17 at day 15 is the main outcome measure, the study is ongoing (as of February 2022)	Phase III, NCT04442503
Dichtel et al. (2020)	Ganaxolone (CCD1042) as augmentation strategy, open-label, pilot study, *N* = 10, MDD with insufficient response, 8 weeks	MADRS scores decreased during 7 weeks, 44% response rate at week 8. Sleep quality, appetite changes, and body weight also improved. Sleepiness, fatigue, and dizziness were the most common adverse events	N/A, NCT02900092
Marinus Pharmaceuticals (2018)	Ganaxolone i.v., *N* = 58, severe PPD, 34 days	HAMD-17 total score decreased vs placebo at 48 h and the decrease was maintained until day 34. Sedation, dizziness were the most commonly reported adverse events	Phase II, NCT03228394
National Library of Medicine (2021d)	Ganaxolone i.v. 6 h, followed by oral administration 28 days, *N* = 33, PPD	HAMD-17 scores decreased rapidly at 6 h but did not separate zuranolone from placebo at day 28	Phase II, NCT03460756
National Library of Medicine (2021e)	PRAX-114 in MDD patients, DBRCT, *N* = 200 and 125, respectively, 43 days	The change in the HAMD total score at day 15 is the main outcome measure; studies are ongoing (as of February 2022)	Phase II/III, NCT04832425
			Phase II, NCT04969510

CGI-I, Clinical Global Impression- Improvement; DBRCT, double-blind randomized controlled trial; HAMD-17, Hamilton Depression Rating Scale; MADRS, Montgomery-Asberg Depression Rating Scale; MDD, major depressive disorder; NLM, National Library of Medicine; PPD, post-partum depression; SAE, severe adverse event.

## Anti-Inflammatory Therapies as Potential Treatments for Depressive Disorders

Anti-cytokine therapies have been recently cornered as potential strategies for decreasing MDD symptoms severity, although they have been mostly investigated in patients with severe, chronic organic diseases, where the mood manifestations were not the center of clinical attention, but associated features (Drevets et al., 2022). There is overwhelming evidence that immune dysregulation is frequently associated with depression, and MDD has been associated with elevated levels of pro-inflammatory cytokines and acute-phase proteins both in the central nervous system and in the blood, but also with decreased adaptive immune response, a bias toward autoimmunity, and other immune changes (Drevets et al., 2022). The effects of anti-cytokine therapies as adjunctive agents in patients with treatment-resistant MDD or bipolar depression have been investigated in clinical trials, with mixed results (Drevets et al., 2022).

A meta-analysis of the trials investigating the effects of anti-inflammatory cytokines interventions in patients with chronic inflammatory conditions, where depressive symptoms severity was measured as a secondary outcome, showed a significant antidepressant effect vs placebo (based on data from seven randomized controlled studies, with 2370 participants) (Kappelmann et al., 2018). Antitumor necrosis factor (TNF) drugs were the most investigated interventions (*n* = 5 trials) in this meta-analysis, and adalimumab, etanercept, infliximab, and tocilizumab all showed statistically significant improvements in depressive symptoms (Kappelmann et al., 2018). In separate meta-analyses (*n* = 2 randomized controlled trials and eight nonrandomized and/or placebo studies) the results were similar, with small-to-medium effect estimates favoring anti-cytokine therapy (Kappelmann et al., 2018). The baseline symptom severity was associated with predictive value for antidepressant effect, but other variables, like the severity of the physical illness, sex, age, or study duration, did not have predictive value (Kappelmann et al., 2018).

According to a mega-analysis of randomized, placebo-controlled trials (*n* = 18) for one of nine disorders (*N* = 10,743 patients diagnosed with ulcerative colitis, rheumatoid arthritis, psoriasis, asthma, ankylosing spondylitis, multicentric Castleman’s disease, osteoarthritis, lupus, neuropathic pain), patients with high severity scores had modest, but significant effects on core symptoms and quality of life-related measures (mental health and vitality) under immune therapy targeting one of seven mechanisms (IL-6, TNF-α, IL-12/23, CD20, COX2, BLγS, p38/MAPK14) (Wittenberg et al., 2020). Anti-IL6 antibodies and anti-IL-12/23 antibodies had larger effects on depressive symptoms than other drug classes (Wittenberg et al., 2020). Effects of anti-IL-12/23 remained significant and anti-IL-6 antibodies remained only at a trend level of efficacy after controlling for physical response to treatment (Wittenberg et al., 2020).

**Etanercept** was evaluated in a double-blind, randomized trial, with 618 patients diagnosed with moderate to severe psoriasis, in a 50-mg twice-weekly regimen vs placebo (Tyring et al., 2006). After 12 weeks of monitoring, 47% of patients in the active group achieved the primary outcome, i.e. ≥75% improvement from baseline in Psoriasis Area and Severity Index (PASI) score vs 5% in the placebo group (Tyring et al., 2006). Also, a higher proportion of the patients receiving etanercept had ≥50% improvement in HAMD or BDI at week 12 compared to placebo, and the improvement in the fatigue was significant and clinically meaningful at the endpoint (Tyring et al., 2006). Improvements in depressive symptoms were weakly correlated with objective measures of skin clearance or joint pain (Tyring et al., 2006).

In a phase III, randomized, double-blind clinical trial, patients with moderate to severe Crohn’s disease (*N* = 499) **adalimumab** was administered every other week or weekly (two maintenance groups) and was compared to adalimumab induction-only group, using measurements for quality of life, depression severity (self-evaluated), fatigue, pain severity, and inflammatory bowel questionnaires, during 56 weeks (Loftus et al., 2008). After 4-week adalimumab induction therapy, patients experienced significant improvements in all measures related to their quality of life (HR-QOL) (Loftus et al., 2008). Patients who continued active treatment after the induction period therapy in a 40 mg every 2 weeks regimen reported less depression, fewer fatigue symptoms, greater improvements in their irritable bowel symptoms, greater SF-36 physical summary scores, and less abdominal pain from weeks 12 to 56 (Loftus et al., 2008). They also presented a greater SF-36 mental component summary score at week 56 (Loftus et al., 2008). The 40-mg adalimumab weekly regimen also was associated with less depression and fewer fatigue symptoms at week 56 (Loftus et al., 2008).

**Ustekinumab** was evaluated in a randomized trial with patients presenting moderate to severe psoriasis (*N* = 1230) who were monitored for their anxiety, depression, and skin-related quality of life for 12 weeks (Langley et al., 2010). Greater improvements at the last study visit were reported for patients receiving ustekinumab (either 45 or 90 mg) vs placebo on all outcomes, determined by the Hospital Anxiety and Depression Scale (HADS)- Anxiety and Depression subscales, and Dermatology Life Quality Index, all of these changes being statistically significant (Langley et al., 2010).

In a 12-week, randomized, double-blind, placebo-controlled, parallel-group trial, 60 participants diagnosed with bipolar I or II depression, presenting also pretreatment biochemical and/or phenotypic evidence of inflammatory activation, were randomized to receive three intravenous infusions of **infliximab** or placebo, as adjunctive treatment, at baseline and weeks 2 and 6 (McIntyre et al., 2019). The primary efficacy outcome was the change at week 12 compared with the baseline of the MADRS scores (McIntyre et al., 2019). Overall baseline-to-end change in the MADRS total score was observed across treatment × time interaction, but the reduction of symptom severity was not significant at week 12 (McIntyre et al., 2019). Infliximab-treated patients with a childhood history of physical abuse exhibited greater reductions in MADRS scores and higher response rates (McIntyre et al., 2019). Therefore, it seems that although the therapeutic benefit of infliximab is minor in patients with bipolar depression, a subpopulation (i.e. those with physical and/or sexual abuse) may have a significant reduction in depressive symptoms during this treatment vs placebo (McIntyre et al., 2019).

In a double-blind, placebo-controlled, randomized clinical trial, 60 medically stable outpatients with MDD, who were either on a consistent antidepressant treatment regimen (*N* = 37) or medication-free (*N* = 23) for 4 weeks or more, and who were moderately resistant to treatment, received three infusions with infliximab (5 mg/kg) or placebo at baseline, weeks 2 and 6 (Raison et al., 2013). No overall difference in change of HAMD scores between treatment groups across time was detected, but there was a significant interaction between treatment × time × hs-CRP concentration (Raison et al., 2013). Changes in HAMD scores (baseline to week 12) favored infliximab vs placebo if the baseline hs-CRP concentration was greater than 5 mg/L, and favored placebo if this concentration was ≤5 mg/L (Raison et al., 2013). A higher rate of response was also detected in patients with baseline hs-CRP>5 mg/L who received infliximab vs placebo (62 vs 33%), without reaching a statistically significant level (Raison et al., 2013). Baseline concentrations of TNF and its soluble receptors were significantly higher in infliximab-treated responders vs nonresponders, and hs-CRP concentrations decreased significantly from baseline to week 12 in the active treatment group compared to placebo (Raison et al., 2013). Again, immune therapy seems to have efficacy in a certain sub-population, namely patients with high baseline inflammatory biomarkers (Raison et al., 2013).

**Losmapimod** (GW856553) is a p38MAPK inhibitor that was administered in a 7.5-mg b.i.d. dosage for 6 weeks in two randomized, placebo-controlled trials in subjects with MDD and prominent symptoms of loss of energy/interest and psychomotor retardation, who also had rheumatoid arthritis (Inamdar et al., 2014). In one of these studies (*N* = 24 patients), prematurely terminated due to variables related to rheumatoid arthritis, the Bech 6-item depression subscale of HAMD-17 favored losmapimod, but in the other study (*N* = 128) no advantage for losmapimod was detected on the same scale (Inamdar et al., 2014). No significant biomarkers (key cytokines) changes were detected during treatment (Inamdar et al., 2014). Based on the combined data of these two trials, 7.5 mg losmapimod was not effective in patients with MDD and rheumatoid arthritis.

According to a systematic review of preclinical and clinical studies, **sirukumab,** an anti-IL-6 human monoclonal antibody, may have potential benefits in patients with inflammatory disorders and neuropsychiatric disorders (Zhou et al., 2017). In individuals with complex psychiatric disorders, e.g. mood disorders, the most likely to benefit domains with sirukumab are negative valence disturbances (anxiety, depression, ruminations), positive valence disturbances (anhedonia), and general cognitive processes (Zhou et al., 2017). Sirukumab (*N* = 176) and **siltuximab** (*N* = 65), both anti-IL-6 antibodies, have also been shown to be effective in reducing depressive symptoms severity in patients with multicentric Castleman disease or rheumatoid arthritis, even after controlling for symptom severity of primary illness, based on two phase 2, double-blind, placebo-controlled trials (Sun et al., 2017). The improvement in depressive symptoms by siltuximab was positively correlated with the baseline soluble IL-6 receptor level (Sun et al., 2017). The improvement in depressive symptoms was significant over placebo only in the siltuximab study (Sun et al., 2017).

An integrated analysis of three randomized, double-blind, controlled, phase 3 trials focused on the efficacy of **ixekizumab** (a high-affinity monoclonal antibody targeting IL-17A) in patients diagnosed with psoriasis and moderately severe depressive symptoms at baseline (QIDS-SR16 total score ≥11) evidenced at week 12 a significantly greater improvement in their depression severity score (80 mg every 2 weeks vs placebo, or 80 mg every 4 weeks vs placebo) (Griffiths et al., 2017). Higher rates of depressive symptoms remission, and significant hsCRP and PASI (Psoriasis Area and Severity Index) reductions were also reported in patients treated with ixekizumab vs placebo (Griffiths et al., 2017).

**Celecoxib** is a cyclooxygenase-2 (COX-2) inhibitor investigated as an adjuvant treatment in patients with MDD, based on the high levels of prostaglandin E2 (PGE2) levels detected in this disorder (Müller et al., 2006). In a prospective, double-blind, add-on study, 40 patients diagnosed with MDD were randomized to either reboxetine + celecoxib, or to reboxetine + placebo (Müller et al., 2006). After 6 weeks, both groups of patients showed significant improvement in HAMD scores, but celecoxib was associated with significantly greater improvement compared to placebo (Müller et al., 2006). In another trial, 30 female outpatients, diagnosed with first episode of depression, were randomized into two groups, one receiving sertraline + celecoxib 100 mg b.i.d, and the other sertraline + placebo twice daily (Majd et al., 2015). Both groups showed improvement in their depressive symptoms from baseline, but celecoxib was associated with a greater decrease in HAMD scores vs placebo after 4 weeks of treatment (Majd et al., 2015). Response rates were also found to be significantly higher in patients who received celecoxib vs placebo, at week 4 (Majd et al., 2015). At week 8, the differences between the two groups were not significant (Majd et al., 2015). This study suggests that celecoxib may hasten the onset of therapeutic action of sertraline, but the differences in efficacy vs placebo are not persistent.

In yet another trial, randomized, double-blind, placebo-controlled, 40 patients with MDD and HAMD baseline score ≥18 were randomized to celecoxib (200 mg b.i.d) or placebo in addition to sertraline, for 6 weeks (Abbasi et al., 2012). Patients who received celecoxib showed a significantly higher reduction of IL-6 serum concentrations and HAMD scores than the placebo group, and also more response and remission (95 and 35% vs 50 and 5%, respectively) (Abbasi et al., 2012). Baseline serum IL-6 levels were significantly correlated with baseline HAMD scores, and also a significant correlation was observed between the reduction of HAMD scores and the reduction of IL-6 serum levels at week 6 (Abbasi et al., 2012).

Anti-inflammatory agents, both immuno-modulators and COX-2 inhibitors, may represent adjuvant strategies to antidepressants in depressive disorders, as the results of clinical trials seem promising until now (Table 6). Larger trials with MDD patients, and not only depressive associated features in chronic organic diseases, are needed, to validate the efficacy of this approach.

**TABLE 6:** Anti-cytokine therapies and COX-2 inhibitors in the pipeline as add-on agents to antidepressants.

Authors/Trial sponsor	Methodology	Results	Clinical trial phase, trial identifier (if available)
Tyring et al. (2006)	Etanercept, DBRT, *N* = 618, psoriasis + depressive symptoms, 12 weeks	HAMD and BDI improvements in the active group vs placebo	Phase III, NCT00111449
Loftus et al. (2008)	Adalimumab, DBRCT, *N* = 499, Crohn’s disease + depressive symptoms, 56 weeks	HR-QOL improvement (SF-36), depressive symptoms, and fatigue improvements	Phase III, NCT00077779
Langley et al. (2010)	Ustekinumab, DBRCT, *N* = 1230, psoriasis + depressive/anxiety symptoms, 12 weeks	HADS- Anxiety and Depression subscales scores significantly improved	Phase III, NCT00307437
McIntyre et al. (2019)	Infliximab as adjunctive treatment, DBRCT, *N* = 60, BD + inflammatory activation, 12 weeks	MADRS’s total score baseline-to-end change was not significant. A higher response rate under infliximab was observed if a childhood history of physical abuse was present	Phase II, NCT02363738
Raison et al. (2013)	Infliximab ± antidepressant, DBRCT, *N* = 60 outpatients, MDD, 12 weeks	HAMD did not record significant changes, but baseline hs-CRP>5 mg/L improved more under infliximab vs placebo	Phase IV, NCT00463580
Inamdar et al. (2014)	Losmapimod (GW856553), DBRCT, *N* = 24 MDD or 128 MDD (two studies), 6 weeks	The first study Bech 6-item subscale of HAMD-17 score evolution favored losmapimod. Study prematurely terminated. The second study no advantage of losmapimod, using the same main outcome measure	Phase II, NCT00569062 Phase II, NCT00976560
Sun et al. (2017)	Sirukumab (CNTO136) and siltuximab (CNTO328), two DBRCT, *N* = 176 methotrexate-resistant rheumatoid arthritis, and 79 multicentric Castleman’s disease, respectively, plus prevalent depressed mood and anhedonia, 12 weeks (sirukumab) or 15 weeks (siltuximab)	SF-36 items for depressive symptoms showed significant improvement only during siltuximab treatment. These improvements were correlated with baseline soluble IL-6 receptor levels	Phase II, NCT00718718
			Phase II, NCT01024036
Griffiths et al. (2017)	Ixekizumab, DBRCT, three studies, psoriasis + depressive symptoms, 12 weeks	QIDS-SR scores reflected a greater improvement in their depression severity score vs placebo. Higher remission rates and significant hsCRP reduction in active groups vs placebo	Phase III, NCT01474512
			Phase III, NCT01597245
			Phase III, NCT01646177
Müller et al. (2006)	Celecoxib + reboxetine/placebo, DBRCT, *N* = 40, MDD, 6 weeks	HAMD scores improved in both groups, but celecoxib outperformed the placebo	Phase IV
Majd et al. (2015)	Celecoxib + sertraline/placebo, DBRCT, *N* = 30, outpatients with first episode of depression, 8 weeks	HAMD scores improved in both groups, with a trend to superiority for celecoxib at week 4, but not at week 8	Phase III, IRCT201009043106N3
Abbasi et al. (2012)	Celecoxib + sertraline/placebo, *N* = 40, MDD, 6 weeks	Celecoxib decreased significantly more IL-6 serum concentrations and HAMD scores vs placebo	Phase I, IRCT138903124090N1

BD, bipolar depression; BDI, Beck Depression Inventory; DBRCT, double-blind randomized controlled trial; HAMD-17, Hamilton Depression Rating Scale; HR-QOL, Health-related quality of life; HADS, Hospital Anxiety Depression Scale; MADRS, Montgomery-Asberg Depression Rating Scale; MDD, major depressive disorder; QIDS-SR, Quick Inventory of Depressive Symptomatology – Self-rated.

**FIGURE 3:**
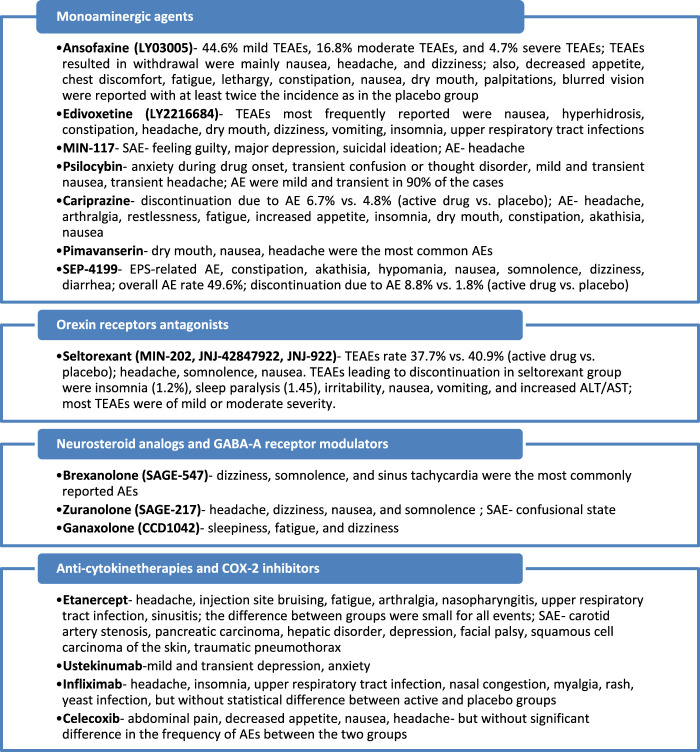
Main adverse events reported in clinical trials for investigational antidepressants. TEAE, treatment-emergent adverse events; AE, adverse events; SAE, severe adverse events; EPS, extrapyramidal symptoms. Based on the data from Mi et al., 2021; Ball et al., 2015; Carhart-Harris et al., 2016; COMPASS, 2021; Citrome, 2019; Fava et al., 2018; Durgam et al., 2016; Fava et al., 2019; Loebel et al., 2022; Savitz et al., 2021; Kanes S. et al., 2017; Gunduz-Bruce et al., 2019; Deligiannidis et al., 2021; Dichtel et al., 2020; Tyring et al., 2006; Langley et al., 2010; Raison et al., 2013; Abbasi et al., 2012.

A synthesis of the safety and tolerability profile of the investigational products reviewed in this article is presented in Figure 3.

## Conclusion

A large number of investigational products with antidepressant properties exist in the pipeline. The monoaminergic hypothesis of depression is still able to generate new drug research, and seven new molecules have been found in phase I to III clinical trials. Besides new drugs (i.e., edivoxetine, ansofaxine, MIN-117, SEP-4199), there are also several already marketed agents that are repurposed for MDD treatment (i.e., cariprazine, pimavanserin), or old psychoactive substances (i.e., psilocybin) tested as add-ons to current antidepressant therapy. Orexin receptor modulators are also investigated for MDD treatment (i.e., seltorexant), with promising results in phase IIb trials (NLM, NCT04951609). Fueled by the success of brexanolone, approved by the FDA for post-partum depression, four new drugs with GABA-A receptors modulating properties/neurosteroids analogs are investigated in phase I to III clinical trials. Anti-cytokine therapies and COX2-inhibitors have been proven to possess antidepressant properties in phase I to IV clinical trials, although not all these studies had positive results. Also, the tolerability of biological therapies should be weighed against their potential benefits. In conclusion, there are promising molecules that had been associated with favorable results in clinical research, but it is difficult to predict which of these agents will be approved in the next few years.

A second part of this review will extend the knowledge regarding new antidepressants in the pipeline, by including drugs with glutamatergic, cholinergic, sestrinergic, and other mechanisms of action.

Regarding the limitations of this review, it should be mentioned that due to the inclusion and exclusion criteria there is a possibility that not all investigational drugs with antidepressant properties were analyzed. Also, the current status of the development for most of the reviewed products was not assessed, but this is related to the lack of this kind of information in the searched databases. Even when manufacturers’ websites were included in the search for new antidepressant drugs, this type of information was generally not available; therefore, it was preferred not to include it in this review.

## Data Availability

The original contributions presented in the study are included in the article/Supplementary Material, further inquiries can be directed to the corresponding author.
