# Supplementary material for: Investigational Drugs for the Treatment of Depression (Part 1): Monoaminergic, Orexinergic, GABA-Ergic, and Anti-Inflammatory Agents
Source: Front Pharmacol. 2022 Jun 14;13:884143. doi: 10.3389/fphar.2022.884143 (PMC9237478; doi:10.3389/fphar.2022.884143)
Supplement: Supplementary file 1 [file DataSheet1.PDF]

Fig.1. Results of the PRISMA-based search paradigm

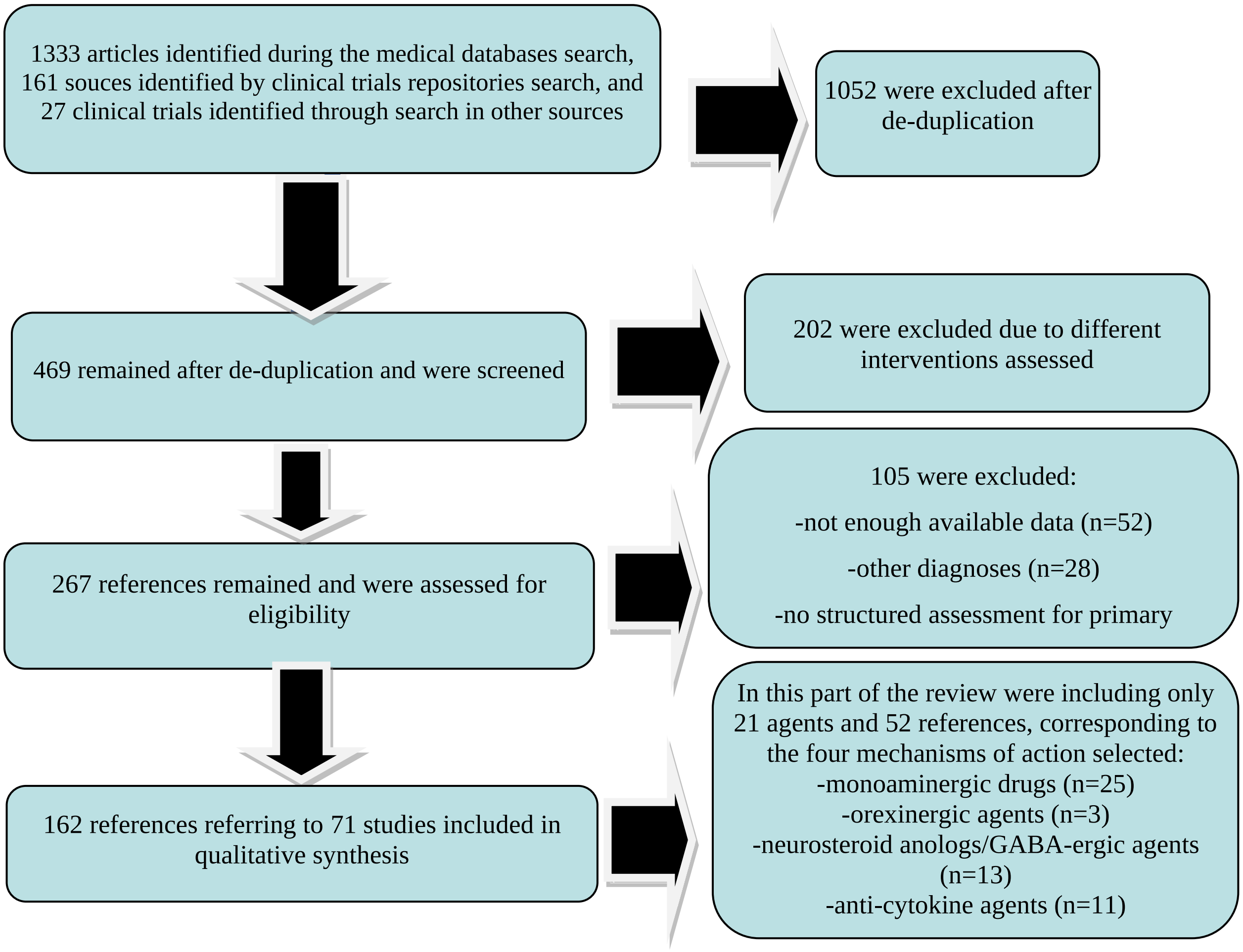

Fig.2. PRISMA-P 2015 Checklist (Moher et al., 2015)

This checklist has been adapted for use with protocol submissions to Systematic Reviews from Table 3 in Moher D et al: Preferred reporting items for systematic review and meta-analysis protocols (PRISMA-P) 2015 statement. Systematic Reviews 2015 4:1

**Section/topic**	**#**	**Checklist item**	**Information reported**		**Line number(s)**
			**Yes**	**No**	
**ADMINISTRATIVE INFORMATION**					
**Title**					
**Identification**	1a	Identify the report as a protocol of a systematic review	☒	☐	68-74
**Update**	1b	If the protocol is for an update of a previous systematic review, identify it as such	☐	☐	Not applicable
**Registration**	2	If registered, provide the name of the registry (e.g., PROSPERO) and registration number in the Abstract	☐	☐	Not applicable
**Authors**					
**Contact**	3a	Provide the name, institutional affiliation, and e-mail address of all protocol authors; provide the physical mailing address of the corresponding author	☒	☐	4-9
**Contributions**	3b	Describe contributions of protocol authors and identify the guarantor of the review	☐	☐	Not applicable, only one author
**Amendments**	4	If the protocol represents an amendment of a previously completed or published protocol, identify it as such and list changes; otherwise, state a plan for documenting important protocol amendments	☐	☐	Not applicable
**Support**					
**Sources**	5a	Indicate sources of financial or other support for the review	☒	☐	755
**Sponsor**	5b	Provide a name for the review funder and/or sponsor	☐	☐	Not applicable
**Role of sponsor/funder**	5c	Describe roles of funder(s), sponsor(s), and/or institution(s), if any, in developing the protocol	☐	☐	Not applicable
**INTRODUCTION**					
**Rationale**	6	Describe the rationale for the review in the context of what is already known	☒	☐	14-63
**Objectives**	7	Provide an explicit statement of the question(s) the review will address concerning participants, interventions, comparators, and outcomes (PICO)	☒	☐	64-66, table 1
**METHODS**					
**Eligibility criteria**	8	Specify the study characteristics (e.g., PICO, study design, setting, time frame) and report characteristics (e.g., years considered, language, publication status) to be used as criteria for eligibility for the review	☒	☐	68-96, table 1
**Information sources**	9	Describe all intended information sources (e.g., electronic databases, contact with study authors, trial registers, or other grey literature sources) with planned dates of coverage	☒	☐	69-70, 77-80
**Search strategy**	10	The present draft of the search strategy is to be used for at least one electronic database, including planned limits, such that it could be repeated	☒	☐	68-96
STUDY RECORDS					
**Data management**	11a	Describe the mechanism(s) that will be used to manage records and data throughout the review	☒	☐	92-96
**Selection process**	11b	State the process that will be used for selecting studies (e.g., two independent reviewers) through each phase of the review (i.e., screening, eligibility, and inclusion in meta-analysis)	☒	☐	68-74, Table 1
**Data collection process**	11c	Describe the planned method of extracting data from reports (e.g., piloting forms, done independently, in duplicate), and processes for obtaining and confirming data from investigators	☒	☐	82-84
**Data items**	12	List and define all variables for which data will be sought (e.g., PICO items, funding sources), any pre-planned data assumptions, and simplifications	☒	☐	Table 1
**Outcomes and prioritization**	13	List and define all outcomes for which data will be sought, including prioritization of main and additional outcomes, with rationale	☒	☐	Table 1
**Risk of bias in individual studies**	14	Describe anticipated methods for assessing the risk of bias of individual studies, including whether this will be done at the	☒	☐	82-84

		outcome or study level, or both; state how this information will be used in data synthesis			
DATA					
**Synthesis**	15a	Describe criteria under which study data will be quantitatively synthesized	☐	☒	
	15b	If data are appropriate for quantitative synthesis, describe planned summary measures, methods of handling data, and methods of combining data from studies, including any planned exploration of consistency (e.g., I^2, Kendall's tau)	☐	☒	
	15c	Describe any proposed additional analyses (e.g., sensitivity or subgroup analyses, meta-regression)	☐	☒	
	15d	If quantitative synthesis is not appropriate, describe the type of summary planned	☒	☐	92-96
**Meta-bias(es)**	16	Specify any planned assessment of meta-bias(es) (e.g., publication bias across studies, selective reporting within studies)	☐	☒	
**Confidence in cumulative evidence**	17	Describe how the strength of the body of evidence will be assessed (e.g., GRADE)	☐	☒	

Table 1. Inclusion and exclusion criteria

Operational criteria	Inclusion criteria	Exclusion criteria
**Population**	Selected population groups were allowed- adolescents and adults.<br>No superior age limit was specified.<br>The main diagnoses were major depressive disorder and bipolar depression. Treatment-resistant forms, first mood episodes, or chronic depression were included.<br>Chronic organic co-morbidities were allowed.<br>Diagnoses should be based on criteria specified by the authors of that paper/sponsors of the trial.<br>Both ICD10 and DSM (IV, IV-TR, or 5) diagnosis criteria were allowed.	Studies that did not specify age limits for their samples, and studies that enrolled children.<br>The presence of psychiatric comorbidities with significant impact on cognition, mood, behavior, and overall functionality (e.g., psychotic disorders, severe neurocognitive disorders, substance use disorders).
**Intervention**	Pharmacological, or combined, pharmacological and psychotherapeutic interventions. New investigational drugs, or repurposed drugs for antidepressant use were included.<br>Only monoaminergic, orexinergic, GABA-ergic/neurosteroids, and anti-inflammatory agents are included in this part of the review.	Psychotherapy as monotherapy for MDD/bipolar depression.<br>Already marketed antidepressants, FDA-approved for all the indications specified in the „population” section of this table, if they were the main intervention. These types of agents were allowed only as active comparators.
**Environment**	Both in-patient and out-patient regimens.	Unspecified environment.
**Primary and secondary variables**	Evaluation of the efficacy, safety, and tolerability of new investigational drugs with antidepressant properties.	All research with unspecified variables.<br>Reviews without pre-defined quantifiable objectives, or poorly defined primary outcome measures.
**Study design**	Any phase of clinical investigation, from I to III was admitted if it corresponded to the pre-defined objective of this review.<br>Phase IV studies were permitted, if specific variables related to depression were included, for drugs not approved for this indication.	Studies with unspecified or poorly defined design. Studies with unclearly defined population/ statistical methods.<br>Case reports, case series.

**Language**	Any language of publication was admitted if the *in-extenso* published paper was available.<br>The same language criteria were applied for clinical trials identified in metadata repositories.	

Fig.3. Mechanisms of action of the identified antidepressants in the pipeline, which are presented in this review

Monoaminergic agents

- **Ansofaxine (LY03005)**- triple reuptake inhibitor of serotonin, norepinephrine, and dopamine
- **Edivoxetine (LY2216684)**- norepinephrine reuptake inhibitor
- **MIN-117**- α1A, α1B, 5HT1A, and 5HT2A receptors modulatorOrexin receptor modulators
- **Psilocybin**- 5HT2A receptor antagonist
- **Cariprazine**- atypical antipsychotic with D3/D2 and 5HT1A receptors partial antagonist properties
- **Pimavanserin**- 5HT2A receptor antagonist/inverse agonist, and, to a lesser extenst, 5HT2C receptor antagonist/inverse agonist
- **SEP-4199**- non-racemic amisulpride, with higher affinity for 5HT7 receptors than D2 receptors

Orexin receptors antagonists

- **Seltorexant (MIN-202, JNJ-42847922, JNJ-922)**- selective OX2 receptors antagonist

Neurosteroid analogs and GABA-A receptor modulators

- **Brexanolone (SAGE-547)**- allopregnanolone, positive allosteric modulator of the synaptic and extrasynaptic GABA-A receptors
- **Zuranolone (SAGE-217)**- neuroactive steroid, GABA-A receptor positive allosteric modulator
- **Ganaxolone (CCD1042)**- allopregnanolone analog, positive allosteric modulator of GABA-A receptors
- **PRAX-114**- GABA-A receptor pozitive allosteric modulator, mainly for extrasynaptic receptors

Anti-cytokinetherapies and COX-2 inhibitors

- **Etanercept**- Tumor necrosis factor (TNF) inhibitor
- **Adalimumab**- human IgG1 monoclonal antibody that binds TNFα
- **Ustekinumab**- human IgG1 monoclonal antibody with anti-IL12 and IL-23 properties
- **Infliximab**- chimeric (human and murine) IgG1 monoclonal antibody that binds TNFα
- **Losmapimod**-selective p38α/β mitogen-activated protein kinase inhibitor
- **Ixekizumab**- humanized IgG monoclonal antibodies that binds selectively to IL-17A
- **Celecoxib**- selective COX-2 inhibitor

Table 2. Monoaminergic modulators with antidepressant properties in the pipeline

Authors/ Trial sponsor	Methodology	Results	Clinical trial phase, trial identifier (if available)
Mi et al., 2021	Ansofaxine (LY03005), DBRCT, N=255, MDD, 6 weeks	HAMD-17 total score changes at week 6 were significant vs. placebo. The overall tolerability was good.	Phase II, NCT03785652
Luye Pharma, 2022; NLM, 2021	Ansofaxine, DBRCT, N=58, MDD, 8 weeks	MADRS total score, HAMD-17 total score, CGI, HAMA, HAMD-17 Anxiety/Somatization factor, Cognitive Impairment factor, Blocking factor, MADRS Anhedonia factor, SDS total score- all were statistically significant improved vs. placebo at week 8. No SAE occurred during this trial. Nausea, vomiting, headache, and drowsiness were the most commonly reported adverse events.	Phase III, NCT04853407
Ball et al., 2016	Edivoxetine (LY2216684) adjunctive to the ongoing antidepressant regimen, three DBRCT, N=701, 689, and 449, MDD with partial response to SSRI, 8 weeks	The mean outcome was the mean change from baseline to week 8 in the MADRS total score. This outcome was not reached by any of these 3 trials. Most of the secondary objectives were not reached, either.	Phase III, NCT01173601 Phase III, NCT01187407 Phase III, NCT01185340
Oakes et al., 2015	Edivoxetine, N=1249, MDD, 8 weeks open-label (edivoxetine + SSRI) + open-label 12 weeks stabilization period + DBRCT 24 weeks	No significant difference between edivoxetine and placebo was detected at the end of the trial (evaluated by MADRS total score).	Phase III, NCT01299272
Ball et al., 2014	Edivoxetine /placebo adjunctive to SSRI, DBRCT, N=131, MDD partial responsive to SSRI, 10 weeks	No significant differences in efficacy between groups at the end of the trial, based on the MADRS total score.	Phase II, NCT00840034
Pangallo et al., 2011	Edivoxetine, DBRCT, N=495, MDD, 10 weeks	MADRS scores were improved significantly by edivoxetine vs. placebo at week 10. Higher rates of response and remission were higher with edivoxetine. SDS scores also were significantly improved vs. placebo.	Phase II/III, NCT00795821
Ball et al., 2015	Edivoxetine as adjunctive to SSRI, open-label, N=328, MDD with partial response to SSRI, 54 weeks	The study discontinuation rate due to adverse events was 17%, 13 SAE (1 death). Most commonly reported adverse events: nausea, hyperhidrosis, constipation, headache, dry mouth, dizziness, vomiting, insomnia, upper respiratory tract infection. Mean MADRS score improvements were -17.0 at week 54.	Phase III, NCT01155661

Davidson et al., 2016	MIN-117 vs. placebo vs. paroxetine, DBRCT, N=84, moderate-to-severe MDD, 6 weeks	MADRS total score was improved by MIN-117 vs. placebo at week 6. Remission with MIN-117 was achieved by 24% of patients (2.5 mg investigational product). The overall tolerability was good.	Phase II, EudraCT 2015-000306-18
NLM, 2022	MIN-117, DBRCT, N=360, adult MDD patients, 6 weeks	No significant differences between active drug and placebo were detected by MADRS, HAMA, and CGI-S scores evolution.	Phase II, NCT03446846
Carhart-Harris et al., 2004	Psilocybin vs. escitalopram, DBRCT, N=59, moderate-to-severe MDD, 6 weeks	QIDS-SR scores at week 6 were not significantly changed vs. placebo. Response rate 70% (psilocybin) vs. 48% (placebo).	Phase II, NCT03429075
Griffiths et al., 2016	Psilocybin, DBRCT, cross-over trial, N=51 cancer patients + depression + anxiety, 5 weeks + 6 months follow-up	GRID-HAMD-17 and HAM-A scores were decreased by high-dose psilocybin. Quality of life, life meaning, and optimism scores improved, and death anxiety decreased under psilocybin treatment. At 6 months these changes persisted, 80% of these patients presented clinically significant decreases in depressed mood and anxiety scores.	Phase II, NCT00465595
Ross et al., 2016	Psilocybin vs. niacin + psychotherapy, DBRCT, N=29 patients with cancer-related anxiety and depression, 7 weeks, cross-over design	Rapid and sustained improvements in anxiety and depression before crossover, plus decreases in cancer-related demoralization and hopelessness, improvements in spiritual well-being, and quality of life. At the follow-up visit (6.5 months) consistent anxiolytic and antidepressant effects were present in the psilocybin group.	Phase I, NCT00957359
Carhart-Harris et al., 2016	Psilocybin + psychological support, open-label, N=12, moderate-to-severe, treatment-resistant MDD, 3 months	The mean self-rated intensity of psilocybin effects was dose-related, and the drug was well tolerated by all patients. Depressive symptoms were markedly reduced at 1 week and 3 months compared to baseline, after high-dose treatment. Anhedonia and anxiety were markedly improved, also.	Phase II, ISRCTN14426797
Davis et al., 2021	Psilocybin, DBRCT, N=24, MDD + psychotherapy, 4 weeks	The mean GRID-HAMD scores were significantly lower in the immediate treatment group, and the QIDS-SR scores reflected a rapid decrease in mean depression score after the first session, which remained significant up to week 4. In the overall sample, 71% of the participants had week 1 and week 4 clinically significant responses to the intervention. The remission rate was 58% at week 1 and 54% at week 4.	Phase II, NCT03181529
COMPASS, 2021	Psilocybin + psychological support, DBRCT, N=233, treatment-resistant MDD, 4 weeks	The high dose drug (25 mg) induced a significant decrease in MADRS scores vs. inactive dose after day 1, and these improvements persisted after week 3, but the difference between the low dose (10	Phase IIb, NCT03775200

		mg) group and the control group was not significant.	
Fava et al., 2018	Cariprazine (low doses/high doses) adjunctive to antidepressant, DBRCT, N=231, treatment-resistant MDD, 19 weeks	No differences were reported on any measures between low doses of cariprazine and placebo, and higher doses led to numerically greater mean change in MADRS and CGI-I scores. MADRS response and remission rates were higher vs. placebo, but without reaching statistical significance. The overall tolerability was good.	Phase II, NCT00854100
Durgam et al., 2016	Cariprazine (low doses/high doses) adjunctive to antidepressants, DBRCT, N=269, treatment-resistant MDD, 8 weeks	Reductions in MADRS total score at week 8 was significantly greater for the high dose of cariprazine vs. placebo, but not for the low dose. Treatment-emergent adverse events most commonly reported were akathisia, insomnia, and nausea.	Phase II, NCT01469377
Earley et al., 2018	Cariprazine adjunctive to antidepressants, DBRCT, N=530, 8 weeks	Cariprazine did not significantly improve MADRS total score or SDS score vs. placebo. A non-significant decrease of depressive symptoms was, however, recorded in the cariprazine-treated patients vs. placebo group. Cariprazine improved significantly CGI-I score vs. placebo, and a significantly higher proportion of patients achieved MADRS response with cariprazine vs. placebo (but not significant). The overall tolerability of cariprazine was good.	Phase III, NCT01715805
Fava et al., 2019	Pimavanserin as an adjunctive agent, DBRCT, N=207, MDD with inadequate response to SSRI/SNRI, 10 weeks	Pimavanserin + ongoing SSRI/ SNRI treatment significantly improved depressive symptoms (reflected in HAMD-17 total score change). Dry mouth, nausea, and headache were the most common adverse events in pimavanserin-treated patients.<br>In patients with anxious depression, the response rate was 55.2% vs. 22.4% (pimavanserin vs. placebo) and the remission rate was 24.1% vs. 5.3% (pimavanserin vs. placebo), among patients with a baseline Anxiety/Somatization factor $\geq$7.	Phase II, NCT03018340
NLM, 2019	Pimavanserin as adjunctive agent DBRCT, N=298, MDD with inadequate response to antidepressant treatment, 5 weeks	Recruitment incomplete due to COVID-19-related problems. A 9 points HAMD total score decline at week 5 for pimavanserin treatment was reported vs. 8.1 points for placebo (p=0.295). A CGI-S change at week 5 of -1.4 vs. -1.1 (pimavanserin vs. placebo) was also reported. Response and remission rates were 31.1% and 18.2% vs. 30.9% and 16.8% (pimavanserin vs. placebo).	Phase III, NCT03968159

NLM, 2019	Pimavanserin as an adjunctive agent, N=236, MDD and inadequate response to antidepressant treatment, 52 weeks	The trial was prematurely terminated „for business reasons and not due to safety concerns".	Phase III, NCT04000009
Loebel et al., 2022	SEP-4199, DBRCT, N=289/337 patients, BD type I, 6 weeks	Endpoint improvement in MADRS total score was observed on both the primary analysis (N=289 participants) for SEP-4199 200 mg/day and 400 mg/day and the secondary, full ITT, analysis (N=337 participants) for both regimens. Median increases in prolactin were +83.6 µg/L for the 200 mg/day dosage, +95.2 µg/L for 400 mg/day.	Phase II, NCT03543410
NLM, 2021	SEP-4199, DBRCT, N=522 (estimated), BD type I, 6 weeks	The trial is ongoing.	Phase III, NCT05169710

BD= bipolar depression; CGI-I= Clinical Global Impression- Improvement; CGI-S= Clinical Global Improvement-Severity; DBRCT= double-blind randomized controlled trial; HAMA= Hamilton Anxiety Rating Scale; HAMD-17= Hamilton Depression Rating Scale; MADRS= Montgomery-Asberg Depression Rating Scale; QIDS-SR= Quick Inventory of Depressive Symptomatology - Self-rated; MDD= major depressive disorder; NLM= National Library of Medicine; SAE= severe adverse event; SDS= Sheehan Disability Scale; SNRI= Serotonin and norepinephrine reuptake inhibitor; SSRI= Selective serotonin reuptake inhibitor

Table 3. Orexinergic agents with antidepressant properties in the pipeline

**Authors/ Trial sponsor**	**Methodology**	**Results**	**Clinical trial phase, trial identifier (if available)**
Recourt et al., 2019	Seltorexant (MIN-202, JNJ-42847922, JNJ-922) vs. diphenhydramine vs. placebo, DBRCT, N=47, MDD, 4 weeks	Core symptoms of depression were improved after 10 days with seltorexant vs. placebo and its efficacy persisted up to day 28.	Phase Ib, NCT02476058
Savitz et al., 2021	Seltorexant + ongoing antidepressant, DBRCT, N=287, MDD with insufficient response to 1-3 SSRI/SNRI, 6 weeks	MADRS scores improved more in the seltorexant (20 mg) vs. placebo at weeks 3 and 6. If baseline ISI≥15 the efficacy of seltorexant 20 mg/day was higher vs. placebo.	Phase IIb, NCT03227224
NLM, 2021	Seltorexant + ongoing antidepressant, DBRCT, N=52 (estimated), MDD with inadequate response to SSRI/ psychotherapy	The outcomes will be related to tolerability, depression severity, clinical global impression, sleep quality, cognitive performance, and pharmacokinetic parameters	Phase I, NCT04951609

DBRCT= double-blind randomized controlled trial; ISI= Insomnia Severity Index; MADRS= Montgomery-Asberg Depression Rating Scale; MDD= major depressive disorder; NLM= National Library of Medicine; SNRI= Serotonin and norepinephrine reuptake inhibitor; SSRI= Selective serotonin reuptake inhibitor

Table 4. Neurosteroid analogs and GABA-A receptor modulators with antidepressant properties in the pipeline

Authors/ Trial sponsor	Methodology	Results	Clinical trial phase, trial identifier (if available)
Kanes et al., 2017a	Brexanolone (SAGE-547), open-label, N=4, PPD, 84 hours	Mean HAMD and CGI-I scores had favorable evolution; 14 adverse events were reported, but no SAE	Phase II, NCT02285504
Kanes et al., 2017b	Brexanolone, DBRCT, N=21, severe PPD, 60 hours	HAMD total scores decreased significantly vs. placebo at 60 h. Dizziness and somnolence- were the most frequently reported adverse events.	Phase II, NCT02614547
Meltzer-Brody et al., 2018	Brexanolone, two DBRCT, N=138 and 108, severe PPD, 60 hours	HAMD scores evolution supported the existence of a significant clinical improvement vs. placebo, which persisted up to 30 days. Headache, dizziness, somnolence- were the most commonly reported adverse events	Phase III, NCT02942004 Phase III, NCT02942017
Gerbasi et al., 2021	Brexanolone, post-hoc analysis of 3 trials, N=299, PPD, 30 days	Brexanolone was superior to placebo after 60 hours and 30 days. Higher probability to sustain HAMD-defined remission and CGI-I response vs. placebo at day 30.	Phase II, NCT02614547 Phase III, NCT02942004 Phase III, NCT02942017
Hoffmann et al., 2020	Zuranolone (SAGE-217), two trials, DBRCT, N=108 healthy volunteers (72 and 36, respectively), single ascending dose study and multiple ascending dose study	Safety, tolerability, and pharmacokinetics of SAGE-217. Mild and transient sedation was observed. Most adverse events were reported as mild/moderate intensity. No SAE was reported.	Phase I
Gunduz-Bruce et al., 2019	Zuranolone, DBRCT, N=89, MDD, 14 days	HAMD scores improved significantly vs. placebo, no SAE was reported. Dizziness, headache, nausea, and somnolence were the most common adverse events.	Phase II, NCT03000530
Deligiannidis et al., 2021	Zuranolone, DBRCT, N=153, PPD, 45 days	HAMD scores were improved by zuranolone vs. placebo from day 3, up to day 45. HAMA and MADRS also improved under zuranolone treatment vs. placebo. The	Phase III, NCT02978326

		overall tolerability of zuranolone was good, with one SAE (confusional state).	
NLM, 2020	Zuranolone, DBRCT, N=192, severe PPD, 14 days	HAMD-17 at day 15 is the main outcome measure, the study is ongoing (as of February 2022)	Phase III, NCT04442503
Dichtel et al., 2020	Ganaxolone (CCD1042) as augmentation strategy, open-label, pilot study, N=10, MDD with insufficient response, 8 weeks	MADRS scores decreased during 7 weeks, 44% response rate at week 8. Sleep quality, appetite changes, and body weight also improved. Sleepiness, fatigue, and dizziness were the most common adverse events.	N/A, NCT02900092
NLM, 2018	Ganaxolone i.v., N=58, severe PPD, 34 days	HAMD-17 total score decreased vs. placebo at 48 hours and the decrease was maintained until day 34. Sedation, dizziness- were the most commonly reported adverse events	Phase II, NCT03228394
NLM, 2019	Ganaxolone i.v. 6 h, followed by oral administration 28 days, N=33, PPD	HAMD-17 scores decreased rapidly at 6 hours but did not separate zuranolone from placebo at day 28.	Phase II, NCT03460756
NLM, 2021	PRAX-114 in MDD patients, DBRCT, N=200 and 125, respectively, 43 days	The change in the HAMD total score at day 15 is the main outcome measure; studies are ongoing (as of February 2022)	Phase II/III, NCT04832425 Phase II, NCT04969510

CGI-I= Clinical Global Impression- Improvement; DBRCT= double-blind randomized controlled trial; HAMD-17= Hamilton Depression Rating Scale; MADRS= Montgomery-Asberg Depression Rating Scale; MDD= major depressive disorder; NLM= National Library of Medicine; PPD= post-partum depression; SAE= severe adverse event

Table 5. Anti-cytokine therapies and COX-2 inhibitors in the pipeline as add-on agents to antidepressants

**Authors/ Trial sponsor**	**Methodology**	**Results**	**Clinical trial phase, trial identifier (if available)**
Tyring et al., 2006	Etanercept, DBRT, N=618, psoriasis + depressive symptoms, 12 weeks	HAMD and BDI improvements in the active group vs. placebo	Phase III, NCT00111449
Loftus et al., 2008	Adalimumab, DBRCT, N=499, Crohn’s disease + depressive symptoms, 56 weeks	HR-QOL improvement (SF-36), depressive symptoms, and fatigue improvements	Phase III, NCT00077779
Langley et al., 2010	Ustekinumab, DBRCT, N=1230, psoriasis +	HADS- Anxiety and Depression subscales	Phase III, NCT00307437

	depressive/anxiety symptoms, 12 weeks	scores significantly improved	
McIntyre et al., 2019	Infliximab as adjunctive treatment, DBRCT, N=60, BD + inflammatory activation, 12 weeks	MADRS's total score baseline-to-end change was not significant. A higher response rate under infliximab was observed if a childhood history of physical abuse was present.	Phase II, NCT02363738
Raison et al., 2013	Infliximab+/- antidepressant, DBRCT, N=60 outpatients, MDD, 12 weeks	HAMD did not record significant changes, but baseline hs-CRP>5 mg/L improved more under infliximab vs. placebo	Phase IV, NCT00463580
Inamdar et al., 2014	Losmapimod (GW856553), DBRCT, N=24 MDD or 128 MDD (two studies), 6 weeks	The first study- Bech 6-item subscale of HAMD-17 score evolution favored losmapimod. Study prematurely terminated. The second study- no advantage of losmapimod, using the same main outcome measure.	Phase II, NCT00569062 Phase II, NCT00976560
Sun et al., 2017	Sirukumab (CNTO136) and siltuximab (CNTO328), two DBRCT, N=176 methotrexate-resistant rheumatoid arthritis, and 79 multicentric Castleman's disease, respectively, plus prevalent depressed mood and anhedonia, 12 weeks (sirukumab) or 15 weeks (siltuximab)	SF-36 items for depressive symptoms showed significant improvement only during siltuximab treatment. These improvements were correlated with baseline soluble IL-6 receptor levels.	Phase II, NCT00718718 Phase II, NCT01024036
Griffiths et al., 2017	Ixekizumab, DBRCT, three studies, psoriasis + depressive symptoms, 12 weeks	QIDS-SR scores reflected a greater improvement in their depression severity score vs. placebo. Higher remission rates and significant hsCRP reduction in active groups vs. placebo.	Phase III, NCT01474512 Phase III, NCT01597245 Phase III, NCT01646177
Müller et al., 2017	Celecoxib + reboxetine/ placebo, DBRCT, N=40, MDD, 6 weeks	HAMD scores improved in both groups, but celecoxib outperformed placebo	Phase IV
Majd et al., 2015	Celecoxib + sertraline/ placebo, DBRCT, N=30, outpatients with first episode of depression, 8 weeks	HAMD scores improved in both groups, with a trend to superiority for celecoxib at week 4, but not at week 8	Phase III, IRCT201009043106N3

Abbasi et al., 2012	Celecoxib + sertraline/ placebo, N=40, MDD, 6 weeks	Celecoxib decreased significantly more IL-6 serum concentrations and HAMD scores vs. placebo	Phase I, IRCT138903124090N1

BD= bipolar depression; BDI= Beck Depression Inventory; DBRCT= double-blind randomized controlled trial; HAMD-17= Hamilton Depression Rating Scale; HR-QOL= Health-related quality of life; HADS= Hospital Anxiety Depression Scale; MADRS= Montgomery-Asberg Depression Rating Scale; MDD= major depressive disorder; QIDS-SR= Quick Inventory of Depressive Symptomatology - Self-rated

Fig.4. Main adverse events reported in clinical trials for investigational antidepressants

Monoaminergic agents

- **Ansofaxine (LY03005)**- 44.6% mild TEAEs, 16.8% moderate TEAEs, and 4.7% severe TEAEs; TEAEs resulted in withdrawal were mainly nausea, headache, and dizziness; also, decreased appetite, chest discomfort, fatigue, lethargy, constipation, nausea, dry mouth, palpitations, blurred vision were reported with at least twice the incidence as in the placebo group
- **Edivoxetine (LY2216684)**- TEAEs most frequently reported were nausea, hyperhidrosis, constipation, headache, dry mouth, dizziness, vomiting, insomnia, upper respiratory tract infections
- **MIN-117**- SAE- feeling guilty, major depression, suicidal ideation; AE- headache
- **Psilocybin**- anxiety during drug onset, transient confusion or thought disorder, mild and transient nausea, transient headache; AE were mild and transient in 90% of the cases
- **Cariprazine**- discontinuation due to AE 6.7% vs. 4.8% (active drug vs. placebo); AE- headache, arthralgia, restlessness, fatigue, increased appetite, insomnia, dry mouth, constipation, akathisia, nausea
- **Pimavanserin**- dry mouth, nausea, headache were the most common AEs
- **SEP-4199**- EPS-related AE, constipation, akathisia, hypomania, nausea, somnolence, dizziness, diarrhea; overall AE rate 49.6%; discontinuation due to AE 8.8% vs. 1.8% (active drug vs. placebo)

Orexin receptors antagonists

- **Seltorexant (MIN-202, JNJ-42847922, JNJ-922)**- TEAEs rate 37.7% vs. 40.9% (active drug vs. placebo); headache, somnolence, nausea. TEAEs leading to discontinuation in seltorexant group were insomnia (1.2%), sleep paralysis (1.45), irritability, nausea, vomiting, and increased ALT/AST; most TEAEs were of mild or moderate severity.

Neurosteroid analogs and GABA-A receptor modulators

- **Brexanolone (SAGE-547)**- dizziness, somnolence, and sinus tachycardia were the most commonly reported AEs
- **Zuranolone (SAGE-217)**- headache, dizziness, nausea, and somnolence ; SAE- confusional state
- **Ganaxolone (CCD1042)**- sleepiness, fatigue, and dizziness

Anti-cytokinetherapies and COX-2 inhibitors

- **Etanercept**- headache, injection site bruising, fatigue, arthralgia, nasopharyngitis, upper respiratory tract infection, sinusitis; the difference between groups were small for all events; SAE- carotid artery stenosis, pancreatic carcinoma, hepatic disorder, depression, facial palsy, squamous cell carcinoma of the skin, traumatic pneumothorax
- **Ustekinumab**-mild and transient depression, anxiety
- **Infliximab**- headache, insomnia, upper respiratory tract infection, nasal congestion, myalgia, rash, yeast infection, but without statistical difference between active and placebo groups
- **Celecoxib**- abdominal pain, decreased appetite, nausea, headache- but without significant difference in the frequency of AEs between the two groups

TEAE= treatment-emergent adverse events; AE= adverse events; SAE= severe adverse events; EPS= extrapyramidal symptoms

Based on data from Mi et al., 2021; Ball et al., 2015; Carhart-Harris et al., 2016; COMPASS, 2021; Citrome, 2019; Fava et al., 2018; Durgam et al., 2016; Fava et al., 2019; Loebel et al., 2022; Savitz et al., 2021; Kanes et al., 2017b; Gunduz-Bruce et al., 2019; Deligiannidis et al., 2021; Dichtel et al., 2020; Tyring et al., 2006; Langley et al., 2010; Raison et al., 2013; Abbasi al., 2012
